# Ferroptosis: an important player in the inflammatory response in diabetic nephropathy

**DOI:** 10.3389/fimmu.2023.1294317

**Published:** 2023-12-04

**Authors:** Jialing Li, Luxin Li, Zhen Zhang, Peijian Chen, Haiying Shu, Can Yang, Yanhui Chu, Jieting Liu

**Affiliations:** ^1^ College of Life Sciences, Mudanjiang Medical University, Mudanjiang, China; ^2^ Heilongjiang Key Laboratory of Anti-Fibrosis Biotherapy, Mudanjiang Medical University, Mudanjiang, China; ^3^ School of First Clinical Medical College, Mudanjiang Medical University, Mudanjiang, China

**Keywords:** diabetic nephropathy, ferroptosis, inflammation, oxidative stress, signaling pathway

## Abstract

Diabetic nephropathy (DN) is a chronic inflammatory disease that affects millions of diabetic patients worldwide. The key to treating of DN is early diagnosis and prevention. Once the patient enters the clinical proteinuria stage, renal damage is difficult to reverse. Therefore, developing early treatment methods is critical. DN pathogenesis results from various factors, among which the immune response and inflammation play major roles. Ferroptosis is a newly discovered type of programmed cell death characterized by iron-dependent lipid peroxidation and excessive ROS production. Recent studies have demonstrated that inflammation activation is closely related to the occurrence and development of ferroptosis. Moreover, hyperglycemia induces iron overload, lipid peroxidation, oxidative stress, inflammation, and renal fibrosis, all of which are related to DN pathogenesis, indicating that ferroptosis plays a key role in the development of DN. Therefore, this review focuses on the regulatory mechanisms of ferroptosis, and the mutual regulatory processes involved in the occurrence and development of DN and inflammation. By discussing and analyzing the relationship between ferroptosis and inflammation in the occurrence and development of DN, we can deepen our understanding of DN pathogenesis and develop new therapeutics targeting ferroptosis or inflammation-related regulatory mechanisms for patients with DN.

## 1 Introduction

The incidence of diabetes and its complications is increasing annually worldwide, with the number of people living with diabetes more than doubling globally in the last 20 years. Approximately 30–40% of these patients develop diabetic nephropathy(DN), 50% of whom develop end-stage renal disease (ESRD) (1). DN is one of the most common causes of ESRD. Early detection and effectively controlling risk factors leading to DN can help to delay disease the progression and prolong life (2). According to the World Health Organization, diabetes will become the seventh leading cause of death worldwide by 2030 (3). There are two main types of diabetes: type 1 diabetes mellitus (T1DM) and type 2 diabetes mellitus (T2DM). T1DM is caused by insufficient insulin secretion, and T2DM is caused by insulin resistance (4). Although the pathogeneses of T1DM and T2DM differ, almost all cell types in the kidney, including podocytes, tubular epithelial cells, endothelial cells, and mesangial cells, are damaged to varying degrees in both types due to hyperglycemia, which exacerbates kidney damage and functional decline (5). In addition, the accumulation of glucose and its metabolites activates resident macrophages in the kidney. Resident macrophages play variety roles in diabetic kidney injury by releasing cytokines/chemokines, that alter macrophage recruitment and polarization, and enhance renal cell injury and macrophage-myofibroblast transformation (6). DN pathogenesis is closely related to genetic factors, metabolic disorders, oxidative stress, inflammatory reactions, cytokines, autophagy, and other factors. Each factor contributes to disease occurrence and progression individually and through mutual influence with other factors (7). Therefore, an in-depth exploration of the potential pathogenesis of DN is of substantial significance for identifying therapeutic targets and developing better treatment methods and drugs.

To date, DN treatment has mainly involved correcting diabetic patients’ glucose metabolism disorders and hemodynamic abnormalities. However, clinical studies have demonstrated that these treatment measures can only alleviate part of the pathology of DN (8). Numerous further studies on diabetes pathogenesis have confirmed that the inflammatory response plays a critical role in DN pathogenesis due to glucose metabolism disorders and hemodynamic abnormalities (9). High-glucose states, advanced glycation end products, hemodynamic abnormalities, and oxidative stress all promote the inflammatory response in DN (10). The discovery of the inflammatory mechanism of DN has guided the clinical use of appropriate anti-inflammatory treatments for patients with DN, which have demonstrated certain benefits (11, 12). For example, baricitinib acts as a selective inhibitor of JAK1 and JAK2, and in a clinical trial evaluating type 2 diabetic adults at high risk of progressive DN, baricitinib was found to significantly reduce albuminuria, thereby improving DN (13). Other anti-inflammatory drugs, such as pentoxifylline (PTF) (14), Emapticap pegol (NOX-E36) (15), and CCX140-B (16) have also been found to improve DN in clinical trials.

Ferroptosis is a nonapoptotic form of cell death resulting from intracellular iron accumulation, leading to the buildup of harmful lipid peroxides. Ferroptosis involves gene and protein regulation, and is associated with abnormal lipid reactive oxygen species (ROS) accumulation, ultimately resulting in oxidative stress and cell death (17). Ferroptosis is ubiquitous in the body and is increasingly associated with the physiological and pathological processes of numerous diseases. including kidney (18), liver (19), nervous system (20), lung (21), and other system (22) diseases. In the pathogenesis of T2DM, ferroptosis not only disrupts insulin secretion, damages β-cells, and induces ER stress and ROS production, but also contributes to the development of complications associated with diabetes (23). These complications include DN (24), diabetic retinopathy (25, 26), diabetic cardiomyopathy (27), and diabetic wound healing disorders (27). Furthermore, investigations have demonstrated that ferroptosis is intimately linked to inflammation. Through ferroptosis, cells produce substances that aggressively activate the innate immune system and play a role in controlling cellular inflammation, signal transduction, and cell proliferation. When an inflammatory response hits a specific threshold, high levels of proinflammatory cytokines will be released, causing damage to the body. The activation of various inflammation-related pathways can also lead to the occurrence and development of ferroptosis (28, 29). Diabetic kidney cell damage may result in iron metabolism disorders, including iron overload and deficiency. Iron overload increases the risk of oxidative stress and inflammation, further aggravating kidney injury (30). Therefore, this review summarizes the specific mechanisms of ferroptosis. The pathways related to ferroptosis and inflammation, are explored to clarify their potential connections. Finally, based on the latest research progress in DN, the relationships among ferroptosis, inflammation, and DN are discussed and summarized to deepen our understanding of DN, pathogenesis to help the development of novel therapeutics targeting ferroptosis or inflammation-related regulatory mechanisms for patients with DN.

## 2 Ferroptosis mechanism and key regulatory factors

**Figure 1:**
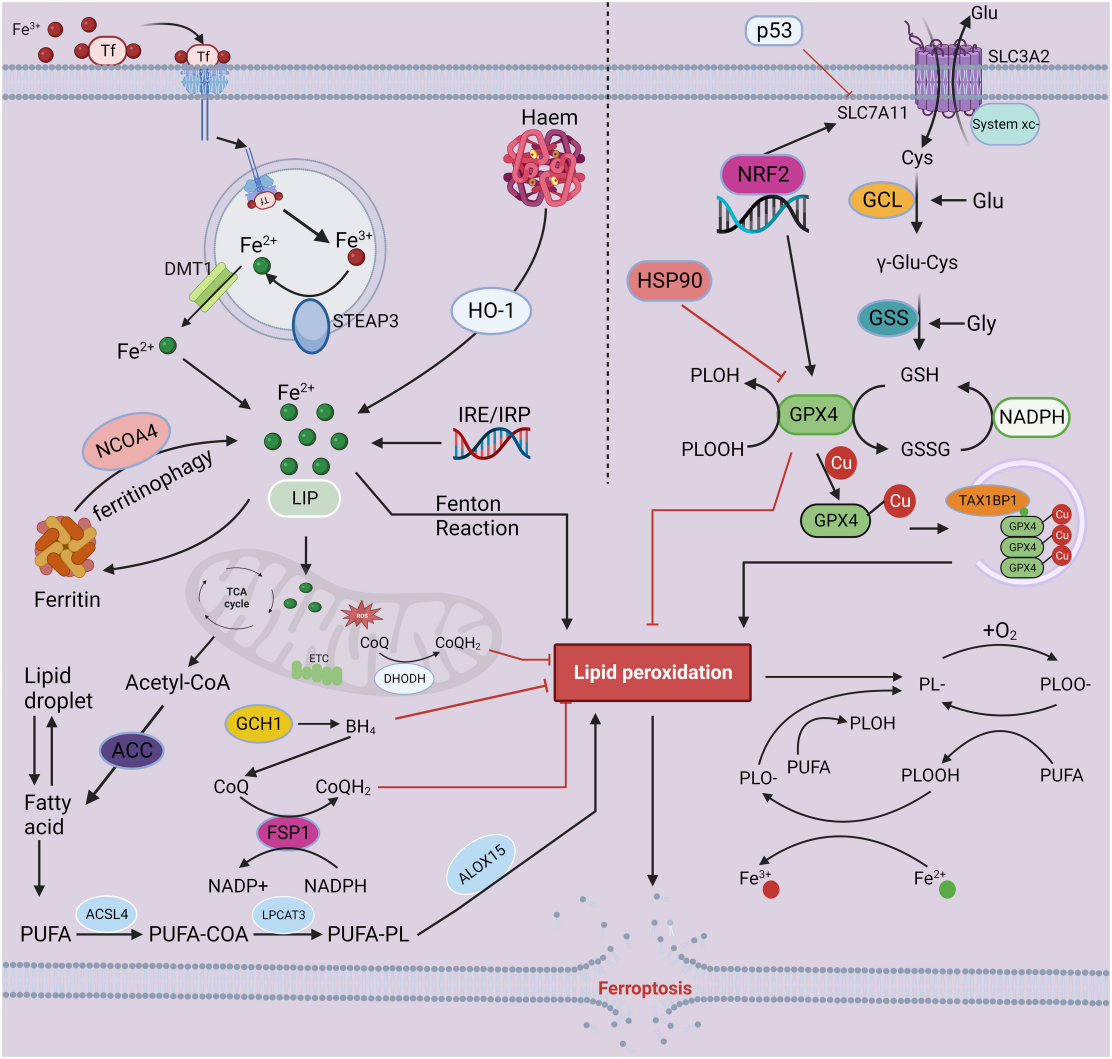
The mechanisms of ferroptosis. Iron-mediated lipid peroxidation is the core process of ferroptosis. Tf carries extracellular Fe^3+^ through its corresponding receptor and transfers it into cells, where Fe^3+^ is reduced to Fe^2+^ by the ferroreductase activity of STEAP3. Finally, DMT1 mediates the release of Fe^2+^ into the cell from the inner body. In addition to entering the nucleus and mitochondria and participating in a range of biochemical reactions, iron may also be stored in ferritin and LIP. As a catalyst, Fe^2+^ converts peroxides to free radicals, such as hydroxyl and hydroperoxyl radicals, through the Fenton reaction, resulting in excessive lipid peroxidation, eventually triggering ferroptosis. Inhibition of the oxidation–reduction system is an important factor in ferroptosis-associated cell death. Currently, four antioxidant pathways have been implicated in ferroptosis: the System X_c_^–^-GSH-GPX4, NADPH-FSP1-CoQ10 regulation, GCH-1-BH4 regulation, and DHODH-mediated mitochondrial ferroptosis defense mechanism pathways. The antitransport system X_c_^-^, containing SLC7A11 and SLC3A2, mediates the uptake of cystine, which is consumed during the synthesis of intracellular GSH. Next, the antioxidant enzyme GPX4 reduces lipid hydroperoxides to lipid alcohols via GSH, protecting cells from ferroptosis. In addition, FSP1, GCH-1 and DHODH can drive the production of antioxidants, such as BH4 and CoQH2, protecting against oxidative stress and ferroptosis. The combined dysregulation of iron metabolism and the oxidation reduction system leads to the accumulation of lipid hydroperoxides in the cell, ultimately leading to ferroptosis. This figure was created with BioRender.

In 2003, Dolma et al. discovered a unique substance called erastin that specifically kills cells expressing small T oncoproteins and oncogenic RAS (31). In 2012, Dixon et al. discovered that desoxamine (DFO)-sensitive ROS buildup occurs during erastin-induced cell death in human cancer cells. This iron-dependent cell death phenotype involves a series of unique morphological, and biochemical features and genetic properties, and is officially referred to as “ferroptosis” (32). Morphologically, during ferroptosis, cells do not show the same morphological characteristics as those undergoing apoptosis (such as chromatin condensation and marginalization), necrosis (such as swelling of the cytoplasm and organelles and rupture of plasma membrane), or autophagy (such as the formation of double-membrane closed vesicles); instead, cells undergoing ferroptosis are characterized by a decrease in mitochondrial volume and crest and an increase in mitochondrial membrane density (32, 33). Biochemically, ferroptosis is primarily characterized by increased intracellular glutathione (GSH) consumption and ROS levels and decreased glutathione peroxidase 4 (GPX4) activity (34). Recent advances in the study of ferroptosis have revealed the mechanisms governing iron metabolism, lipid metabolism, and the System X_c_^–^-GSH-GPX4 pathway, which regulates cellular antioxidants (34–38). In addition, other regulatory pathways and regulatory factors, such as the NADPH-FSP1-CoQ10 regulatory pathway, GCH-1-BH4 regulatory pathway, protein posttranslational modification mechanism, and mitochondrial ferroptosis defense mechanism mediated by dihydro whey acid dehydrogenase (DHODH), have been identified as key factors in ferroptosis (39–49) (**Figure 1**).

### 2.1 Iron metabolism

Iron is necessary for the accumulation of lipid peroxides and ferroptosis (32, 50). Therefore, iron uptake, transport, and storage impact ferroptosis. The maintenance of iron homeostasis in organisms is closely regulated. Iron in the diet is divided into heme and nonheme-bound iron. The absorption site of nonheme iron (Fe^3+^) is mainly in the upper duodenum and jejunum. The absorption of inorganic iron by gastrointestinal mucosal cells is an active process requiring the assistance of many proteins. Iron absorbed by enterocytes can be directly used in intracellular metabolic processes, stored in ferritin, or released into the blood circulation through the basolateral membrane for delivery to other tissues for use and storage. Trivalent iron is first converted to divalent iron by the duodenal cytochrome B (DCYTB) iron reductase found on the parietal membrane of intestinal mucosal cells. Subsequently, reduced iron Fe^2+^ enters enterocytes through divalent metal transporter-1 (DMT1) in the apical membrane (51), is transported into the blood circulation through Ferroportin1 (FPN1) on the intestinal epithelial cell membrane, and is then converted to Fe^3+^ by heparin (Hp) or ceruloplasmin (52). Circulating iron binds to transferrin (Tf) of extracellular trivalent iron (Fe^3+^), binds to the membrane protein transferrin receptor 1 (TFR1) on the cell membrane to form a complex, and enters the cell through endocytosis to form an endosome (53). Fe^3+^ is released from Tf by endosomal acidification and is reduced to ferrous iron (Fe^2+^) through the iron reductase activity of STEAP3. Finally, Fe^2+^ enters the cytoplasm via the iron ion channel DMT1(also known as SLC11A2) in the endosomal membrane (54). In addition to entering the nucleus and mitochondria and participating in a series of biochemical reactions, iron can be stored in ferritin and a labile iron pool (LIP) (54–57). Because iron plays a central role in controlling cell survival and death, intracellular iron homeostasis is finely regulated. Iron regulatory proteins (IRP1 and IRP2) bind to the iron response element (IRE) in the untranslated region (UTR) of the target mRNAs associated with iron metabolism, in turn regulating the production of ferritin or the transferrin receptor involved in iron metabolism and homeostasis (58–61).

Intracellular LIP exists mainly as Fe^2+^. However, due to the instability and high reactive activity of Fe^2+^, many intracellular pathways can alter cells’ sensitivity to ferroptosis by changing the intracellular Fe^2+^ levels. For example, autophagy mediated by nuclear receptor coactivator 4 (NCOA4) can increase intracellular iron levels by degrading ferritin, leading to ferroptosis (62). In addition, heme degradation mediated by heme oxygenase-1 (HO-1) releases iron and is therefore associated with ferroptosis. Numerous previous studies have confirmed that HO-1 demonstrates two functions in ferroptosis induction. In HT1080 fibrosarcoma cells, erastin promotes the production of HO-1, which may encourage ferroptosis (63). Conversely, HO-1 can inhibit ferroptosis in some cell lines (64, 65). Therefore, the specific mechanism of ferroptosis mediated by HO-1 needs further study.

In summary, under the abnormal expression or dysfunction of these iron-related proteins and genes, the increase in intracellular iron concentration leads to an overload of intracellular iron, causing excessive Fe^2+^ ions accumulation. These ions then react with hydrogen peroxide (H_2_O_2_) via the Fenton reaction, generating hydroxyl radicals and highly reactive ROS. The generation of ROS and subsequent hydroxyl radical-mediated lipid peroxidation eventually lead to plasma membrane damage, representing the central events leading to ferroptosis (66).

### 2.2 Lipid metabolism

Polyunsaturated fatty acids (PUFAs) are components of the cell membrane and are responsible for regulating various biological functions (67). Because the C-H bond of the diallyl position in the PUFA structure is weak, it is easily oxidized (68). In addition, membrane PUFAs are the main targets of ROS attack (69). Thus, peroxidation of PUFAs on the cell membrane is the pivotal process underlying ferroptosis. Lysophosphatidylcholine acyltransferase 3 (LPCAT3) and acyl-CoA synthase long-chain family member 4 (ACSL4) are important drivers of lipid peroxidation (70–72). ACSL4 is responsible for catalyzing the connection between long-chain PUFAs and coenzyme A (73), and the products PUFAs and coenzyme A(PUFA-COA) are re-esterified to a fatty acid acyl group (PUFA-PL) by LPCAT3 (74). PUFA-PL is a unique functional lipid group that can provide a substrate for lipid peroxidation and induces ferroptosis (75). The oxidation of PUFA-PL is promoted by nonenzymatic catalysis or enzymatic catalysis, resulting in phospholipid peroxide (PLOOH) formation. The Fenton reaction mediates nonenzymatic lipid peroxidation, while the enzyme-catalyzed reaction is mediated by oxygenase enzymes (ALOXs), particularly ALOX15 (76).

The initiation of lipid peroxidation necessitates the abstraction of diallyl hydrogen atoms positioned between the two carbon-carbon double bonds from polyunsaturated PUFA-PL on phospholipids, resulting in the formation of carbon-centered free radicals (PL-) that subsequently react with molecular oxygen to generate lipid peroxy radicals (PLOO-). These radicals then initiate a cascade by attacking other lipid molecules, capturing their hydrogen atoms and forming new free radicals and lipid hydroperoxide PLOOH. Once PLOOH is formed, if it is not promptly eliminated by GPX4, it gives rise to alkoxy species (PLO-) and PLOO-, facilitated by ferrous ions, which perpetuate destructive peroxy chain reactions (77).

The lipid peroxidation of PUFAs also generates secondary byproducts, including aldehydes, such as malondialdehyde (MDA) and 4-hydroxynonenal (4-HNE), which serve as the primary toxic agents capable of interacting with DNA bases, proteins, and other nucleophilic molecules, leading to profound cytotoxicity (78). The process of ferroptosis induced by lipid peroxides also encompasses alterations in the structure and permeability of cell membranes, impacting cellular survival. At the structural level, PUFAs play a pivotal role in maintaining the integrity of cell membranes, and extensive lipid peroxidation can induce significant alterations in both the chemical composition and geometric arrangement of the lipid bilayer. Additionally, the accumulation of lipid peroxides can induce the formation of membrane pores and disrupt the barrier function, leading to a reduction in membrane thickness and alterations in membrane permeability. In molecular dynamics studies, lipid peroxidation has been shown to increase biofilm curvature, causing the acyl tail of lipid peroxidation (which becomes more hydrophilic) to bend toward the aqueous phase. This structural change leads to membrane instability and subsequent micelle formation, ultimately resulting in altered membrane permeability and impacting cell survival (79).

The production of mitochondrial ROS is also critical for lipid peroxidation and ferroptosis. Mitochondria are the main source of intracellular ROS, in which the superoxide produced by the electron leakage of the electron transport chain (ETC) produces hydroxyl radicals through a series of reactions, thus driving PUFA-PL peroxidation. Electron transport and the mitochondrial proton pump are essential for ATP production and ferroptosis promotion. In ATP deficiency, AMPK is activated and blocks ferroptosis by inhibiting acetyl-CoA conversion to malonyl-CoA (the precursor of PUFA synthesis), mediated by acetyl-CoA carboxylase (ACC). Therefore, multiple mitochondrial metabolic processes are critical drivers of lipid peroxidation (80–83).

The endoplasmic reticulum (ER) is an intracellular membrane system comprising phospholipid molecules, that serve multiple crucial functions in lipid synthesis (84). The ER plays a key regulatory role in lipid metabolism and ROS production during kidney injury (85–87). Stimulated Raman scattering (SRS) microscopic localization imaging revealed that a ferroptosis inhibitor (ferrostatin-1) accumulates in lysosomes, mitochondria, and the ER. However, compounds that modulate ferroptosis by influencing lipid peroxidation are predominantly confined to the ER, implying the pivotal role of the ER in lipid peroxidation during ferroptosis (88). ER viscosity is a critical determinant reflecting its structural integrity, in association with intracellular diffusion, intermolecular interactions, and membrane fluidity. Since the ER is a major base for lipid synthesis and has the largest membrane structure in the cell, it is speculated that the conversion of unsaturated lipids to lipid peroxides during ferroptosis may lead to changes in ER viscosity. Quantitative tracking of ER viscosity during erastin-induced ferroptosis by the TPPLM iridium complex revealed a significant increase in ER viscosity during ferroptosis, revealing the correlation between ER dysfunction and ferroptosis (89). When ER luminal conditions are altered, or chaperone capacity is overwhelmed due to redox state changes, calcium level alterations, or a failure to posttranslationally modify secretory proteins, ER stress occurs, and cells initiate the unfolded protein response (UPR) (90). Ferroptotic agents have recently been demonstrated to elicit ER stress and induce ferroptosis through the ER stress-mediated PERK-eIF2α-ATF4-CHOP signaling pathway (91, 92). In summary, as the central hub driving lipid peroxidation, the ER can lead to cell death through ferroptosis. Therefore, the in-depth study of the ER may be critical in guiding the development of disease treatments in the future.

### 2.3 The system X_c_^–^-GSH-GPX4 pathway

Maintaining cellular redox homeostasis is crucial for determining cell fate. Glutathione peroxidase (GPX), the predominant protein family in the critical redox system of mammalian cells, plays a pivotal role in counteracting oxidative stress and preserving redox equilibrium (93). Among the GPX members, GPX4 is the key regulatory factor in preventing cell ferroptosis (94), and its active center is selenocysteine. GPX4 can reduce intracellular lipid hydroperoxides to nontoxic lipid alcohols, and catalyze the reduction of other organic peroxides, such as hydrogen peroxide (95). GPX4 is a crucial enzymatic regulator of anti-ferroptosis mechanisms and undergoes extensive regulatory control at multiple levels. NFE2L2/NRF2 is a well-established transcription factor that safeguards cells against oxidative stress by orchestrating the upregulation of antioxidant genes, including GPX4 (96). In addition to transcriptional regulation, GPX4 expression can be modulated at the protein level. Heat shock proteins (HSPs) are key regulators of GPX4 protein degradation. For example, HSP90, a ubiquitous HSP, mediates ferroptosis resistance by regulating lysosome-associated membrane protein 2A (LAMP-2A) levels in the chaperone-mediated autophagy (CMA) pathway, which mediates the degradation of GPX4 (97). As a protein chaperone, HSPA5 forms a complex with GPX4, thus inhibiting GPX4 degradation (98). The ubiquitin-proteasome pathway can also mediate GPX4 protein degradation (99). For example, TRIM46 a member of the E3 ubiquitin ligase family, induces ubiquitination and promotes GPX4 degradation, resulting in ferroptosis (25). Some studies have also shown that exogenous copper promotes the ubiquitination of GPX4 and the formation of GPX4 aggregates by selectively binding to cysteine residues C107 and C148 exposed on the surface of the GPX4 protein, while TAX1BP1 (Tax1 binding protein 1) subsequently acts as an autophagy receptor in response to copper stress, promoting GPX4 degradation and subsequent ferroptosis (100). GSH is a powerful reductant and can be used as a cofactor of GPX4 to promote the reduction of PLOOHs to their corresponding alcohols (PLOHs) in cells. Requisite regulation of the GSH axis is imperative for sustaining GPX4 activity (101). GSH can be oxidized to glutathione disulfide (GSSG), while glutathione disulfide reductase (GSR) reconverts GSSG to GSH by the electronic catalysis provided by NADPH/H^+^ (102). Through the cystine/glutamate antiporter (System X_c_^-^), a heterodimer formed by the disulfide bonding of the light chain subunit (SLC7A11) and heavy chain subunit (SLC3A2), intracellular glutamate is exchanged for extracellular cystine at a 1:1 ratio (103), and cystine entering the cells is reduced to cysteine (the most abundant cellular antioxidant) (104). Cysteine serves as a rate-limiting substrate for GSH synthesis (105). Inhibition of this system leads to cellular cysteine depletion and induces ferroptosis (106). The synthesis of GSH requires the participation of cysteine (Cys), glutamic acid (Glu), and glycine (Gly) (107) and involves two steps. First, Glu and Cys are linked by glutamate-cysteine ligase (GCL) to produce the dipeptide γ-glutamylcysteine (γ-Glu-Cys). Next, Gly is added to γ-Glu-Cys by glutathione synthase (GSS) to produce GSH (γ-Glu-Cys-Gly) (108). Most mechanisms by which cellular GSH is reduced depend on sufficient intracellular Cys (109). The intracellular Cys concentration depends on System X_c_^-^. Therefore, the expression of SLC7A11, a key component of the cystine/glutamate antiporter, is critical for ferroptosis (110). NRF2 regulates GSH biosynthesis and related metabolic enzymes, including GCL, GSS, and SLC7A11, all of which are indispensable for GSH synthesis (111). Some tumor suppressor genes, including p53 and interferon, have been demonstrated to inhibit SLC7A11 expression, thereby inhibiting cystine uptake and sensitizing cells to ferroptosis (112–115). Furthermore, the active center of GSH encompasses selenocysteine, implying that selenium axis regulation impacts GPX4 activity (116, 117). Zhang et al. demonstrated that SLC7A11-mediated Cys uptake facilitates GPX4 protein synthesis by activating the rapamycin complex 1 (mTORC1) pathway (118).

However, certain cells can biosynthesize Cys from methionine via the sulfur transfer pathway, thereby conferring a degree of resistance against ferroptosis (119). Additionally, it has been demonstrated that the catalytic subunit of glutamate-cysteine ligase (GCLC) plays a crucial role in preventing intracellular glutamate accumulation during Cys deficiency, thereby effectively inhibiting ferroptosis in non-small cell lung cancer (120).

### 2.4 Other mechanisms

#### 2.4.1 NADPH-FSP1-CoQ10 regulatory pathway

GPX4 is widely considered the main regulator of ferroptosis; however, sensitivity to GPX4 inhibitors varies across cell lines, indicating that other factors are involved (121). Gene inhibitors and CRISPR/Cas9 screening techniques have identified ferroptosis inhibitor protein 1 (FSP1) as the second key factor involved in ferroptosis, independent from the mercaptan-dependent axis. FSP1 has also been found to be an important antioxidant. FSP1 is mainly expressed on the plasma membrane and is an oxidoreductase that reduces ubiquinone (CoQ) to ubiquinol (CoQH2). The reduced form of ubiquinone captures active free radicals and reduces intracellular lipid peroxide production. In addition, FSP1 catalyzes coenzyme Q10 (CoQ10) regeneration through NADPH (39, 40). In conclusion, NADPH-FSP1-CoQ10 functions as an independent parallel system to GPX4, and targeting FSP1 could serve as a promising therapeutic approach to induce cancer cell death.

#### 2.4.2 GCH-1-BH4 regulatory pathway

As the main regulatory pathway for inhibiting ferroptosis, the GCH-1-BH4 axis controls the endogenous production of the antioxidant tetrahydrobiopterin (BH4) (122–124). Tetrahydrobiopterin mitigates lipid peroxidation by facilitating the production of CoQ10, thereby attenuating oxidative damage and inducing lipid remodeling (42). GTP cyclohydrolase 1 (GCH-1) serves as a pivotal regulatory enzyme governing the synthesis of tetrahydrobiopterin, thereby exerting rate-limiting control (124). Overexpression of GCH-1 protects against ferroptosis induced by RSL3, but not apoptosis inducers, indicating that GCH-1 selectively protects cells from ferroptosis (42).

#### 2.4.3 Protein posttranslational modifications

Alterations in protein activity during ferroptosis may be associated with PTMs (125–127). PTMs encompass a range of modifications, such as phosphorylation, acetylation, ubiquitination, methylation, and SUMOylation; Most of these modifications are reversible and play a crucial role in regulating the activity, stability, interactions, and intracellular localization of target proteins. Moreover, they diversify protein functions and serve as pivotal switches that enable cells or organisms to promptly and precisely respond to stressors. For instance, under glucose starvation conditions, AMPK expedites ACC phosphorylation while suppressing PUFA-containing lipid biosynthesis, mediating the inhibitory effect on ferroptosis (128). Recently, a growing emphasis has been placed on the pivotal role of PTMs in ferroptosis (43, 80, 129–131).

#### 2.4.4 Dihydroxy acid dehydrogenase

DHODH is a mitochondrial inner membrane enzyme and the key enzyme in nucleic acid pyrimidine synthesis, catalyzing the fourth step of the pyrimidine biosynthesis pathway. Due to the increasing demand for nucleotides in rapidly proliferating cells, DHODH has been extensively explored as a promising target for cancer therapy in recent years (132–134). DHODH has recently been shown to demonstrate a unique function in reducing mitochondrial lipid peroxidation and ferroptosis independent of its catalytic role in pyrimidine nucleotide synthesis. DHODH inhibits ferroptosis in the mitochondrial inner membrane by reducing CoQ to CoQH2 (135). These results reveal the DHODH-mediated defense mechanism of mitochondrial ferroptosis and demonstrate a potential therapeutic strategy for targeting ferroptosis in cancer.

## 3 Ferroptosis and inflammation

When numerous exogenous and endogenous factors damage cells, tissues, and organs, a series of complex local and systemic reactions, referred to as the inflammatory response, occur to limit and eliminate the damage factors, remove and absorb necrotic tissues and cells, and repair the damage (136, 137). However, inflammatory response dysregulation leads to immune system imbalance, cellular dysfunction, and ultimately mortality. Recent studies have emphasized that inflammation activation, encompassing the stimulation of multiple signaling pathways related to inflammation, can induce ferroptosis (28, 138). As a mechanism of iron-dependent regulation of cell death, ferroptosis exhibits two primary characteristics: intracellular iron overload and disruption of the oxidation-reduction system. The dysregulation of iron metabolism and imbalance in the oxidation-reduction system are pivotal factors contributing to the aberrant inflammatory response (139, 140). Hence, to elucidate the potential association between ferroptosis and inflammation, we next summarize and explore the roles of the inflammation-related cGAS-STING, JAK-STAT, inflammasome, NF-κB, and MAPK pathways in ferroptosis.

### 3.1 cGAS-STING signaling pathway in ferroptosis

**Figure 2:**
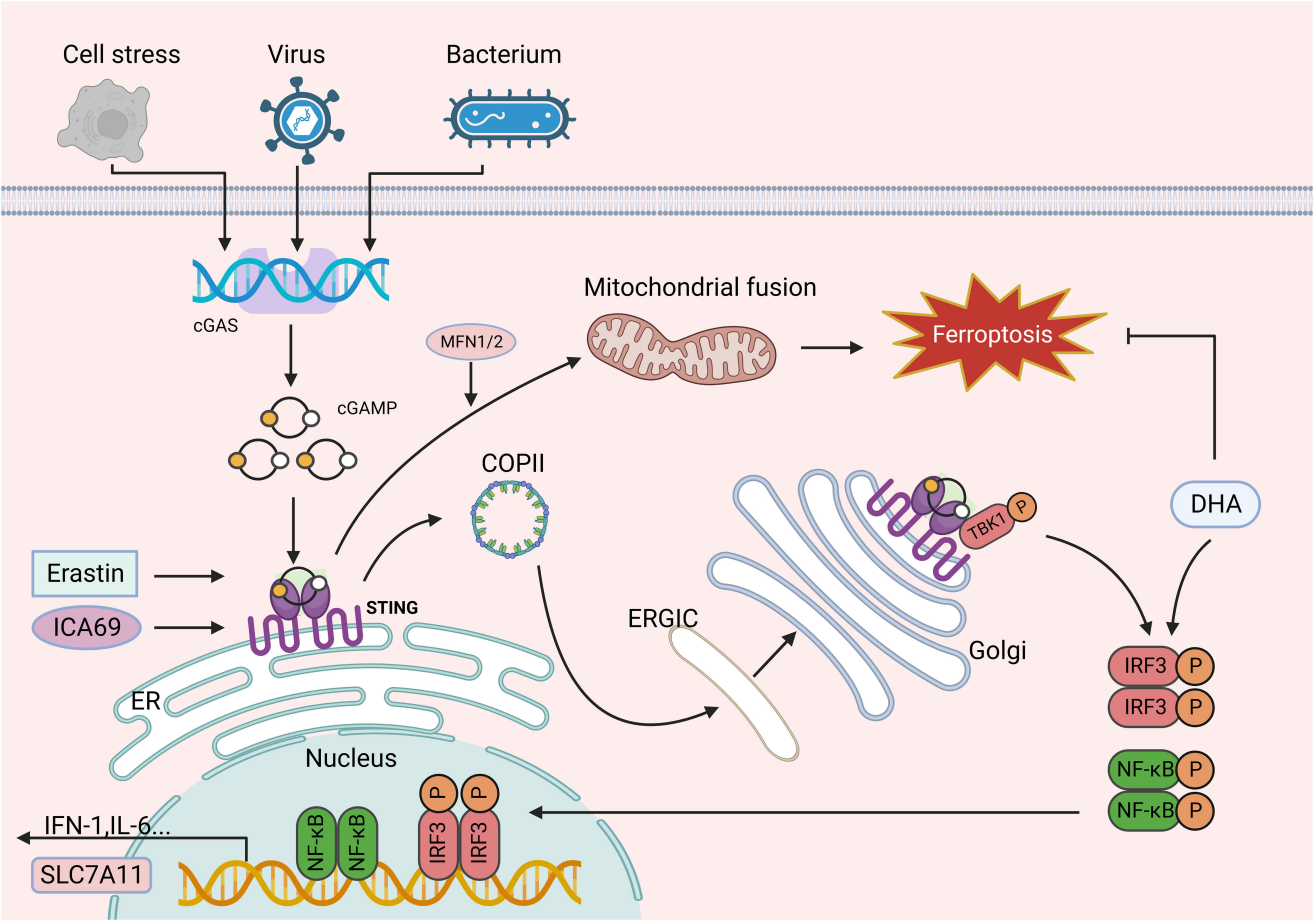
The role of the cGAS-STING signaling pathways in ferroptosis. cGAS catalytic activity is triggered by interaction with dsDNA, and upon binding dsDNA, cGAS dimers assemble on dsDNA, leading to the enzymatic activation of cGAS and cGAMP synthesis. cGAMP binds to STING at the ER membrane for oligomerization and is translocated to the ERGIC and Golgi apparatus, where STING recruits TBK1 and promotes TBK1 autophosphorylation. In turn, STING is phosphorylated by activated TBK1 and further recruits IRF3, which TBK1 phosphorylates. IRF3 phosphorylated by TBK1 dimerizes and translocates to the nucleus, where it induces the expression of IFN, ISG, inflammatory mediator genes, pro-apoptotic genes, and chemokines, which in turn are involved in various pathophysiological processes. Erastin can induce the accumulation of STING in the mitochondria and bind to MFN1/2, triggering mitochondrial fusion and subsequently resulting in ferroptosis. Similarly, increased ICA69 expression triggers STING production, which in turn leads to intracellular lipid peroxidation and ultimately ferroptosis. In addition, as a downstream target of STING, IRF3 is involved in lipid peroxidation and ferroptosis. However, DHA increases IRF3 expression, thereby contributing to SLC7A11 transcription and inhibiting ferroptosis. This figure was created with BioRender.

The cGAS-STING signaling pathway has emerged as a pivotal mediator of inflammation in the context of infection, cellular stress, and tissue injury. The broad involvement of the cGAS-STING pathway is based on its ability to sense and regulate cellular responses to microbial-and host-derived double-stranded DNA (dsDNA), which are ubiquitous danger-associated (DAMP) molecules (141, 142). As a second messenger in the human body, cGAMP is a key molecule in the upstream pathway of STING and plays an important role in the innate immune signaling pathway. In mammals, cGAS catalytic activity is triggered by interaction with dsDNA, when combined with dsDNA, cGAS dimer assembly on dsDNA, causes cGAS enzyme activation and 2 ‘3’ cyclic GMP-AMP (cGAMP) synthesis (143). cGAMP binds to STING at the ER membrane for oligomerization; subsequently, as it passes through the ER-Golgi intermediate compartment (ERGIC) and Golgi apparatus, STING recruitment of TANK binding kinase 1 (TBK1) promotes TBK1 autophosphorylation (141, 144, 145). STING is phosphorylated by activated TBK1, recruiting IFN regulatory factor 3 (IRF3), which TBK1 phosphorylates. Phosphorylation of IRF3 by TBK1 dimerizes and translocates IRF3 to the nucleus, inducing the expression of the type I interferon (IFN) gene, interferon-stimulated genes (ISGs), and several other inflammatory mediators, pro-apoptotic genes, and chemokines, which in turn are involved in various pathophysiological processes (141, 145, 146) (**Figure 2**).

Increasing evidence suggests an interaction between ferroptosis and the cGAS-STING pathway. Ginsenoside Rd inhibits ferroptosis through the cGAS/STING pathway and alleviates CCL4-induced acute liver injury in mice (147). Mn^2+^ increases the phosphorylation levels of STING, IRF3, and TBK1, and upregulated IFN expression via the cGAS-STING signaling pathway. Inhibition of cGAS-STING or type I IFN restores DHODH expression, reverses lipid peroxidation and ROS production, and rescues Mn^2+^-induced ferroptosis in tumor cells (148). Another study revealed that DNA damage-induced STING activation promotes autophagy-dependent ferroptosis in human pancreatic cancer cells (149).

STING is a transmembrane protein typically located in the ER and is a key regulator of innate immunity. STING has been confirmed to be closely related to ferroptosis. The classical ferroptosis inducer erastin has been found to induce the accumulation of STING1 in mitochondria and bind to mitofusin 1/2 (MFN1/2), thereby initiating mitochondrial fusion. This process leads to the subsequent generation of ROS and lipid peroxidation. STING or MFN1/2 knockdown reduces cell sensitivity to ferroptosis and alleviates imidazolone erastin-induced tumor suppression in xenograft models (150). Islet cell autoantigen 69 (ICA69) is another important molecule regulating inflammation and the immune response in many diseases. Increased ICA69 expression triggers STING production, leading to intracellular lipid peroxidation and, ultimately, ferroptosis and cardiac damage (151). IRF3, a downstream target of STING, is also involved in lipid peroxidation and ferroptosis. The regulatory mechanism of docosahexaenoic acid (DHA) in myocardial microvascular endothelial cells in rats with cardiac hypertrophy has been investigated, revealing that DHA prevents microvascular endothelial cell dysfunction and alleviates cardiac dysfunction by inhibiting ferroptosis. In this mechanism, DHA increases IRF3 expression, which contributes to SLC7A11 transcription, thereby reducing arachidonic acid 12-lipoxygenase (ALOX12) production and inhibiting ferroptosis (152). Collectively, these findings demonstrate the impact of activated cGAS-STING signaling on lipid peroxidation and ferroptosis, suggesting that targeting this pathway could alleviate the adverse effects of ferroptosis. Furthermore, Dai et al. reported that a high-iron diet or GPX4 deletion exacerbates pancreatitis by inducing ferroptosis in mice and promotes KRAS-mediated pancreatic tumorigenesis. Enhanced oxidative stress resulting from GPX4 depletion or a high iron diet leads to the release of 8-OHG, an oxidative DNA damage product, that activates the TMEM173/STING-dependent DNA sensor pathway and facilitates macrophage infiltration in pancreatic tissues (149).

### 3.2 JAK-STAT signaling pathway in ferroptosis

**Figure 3:**
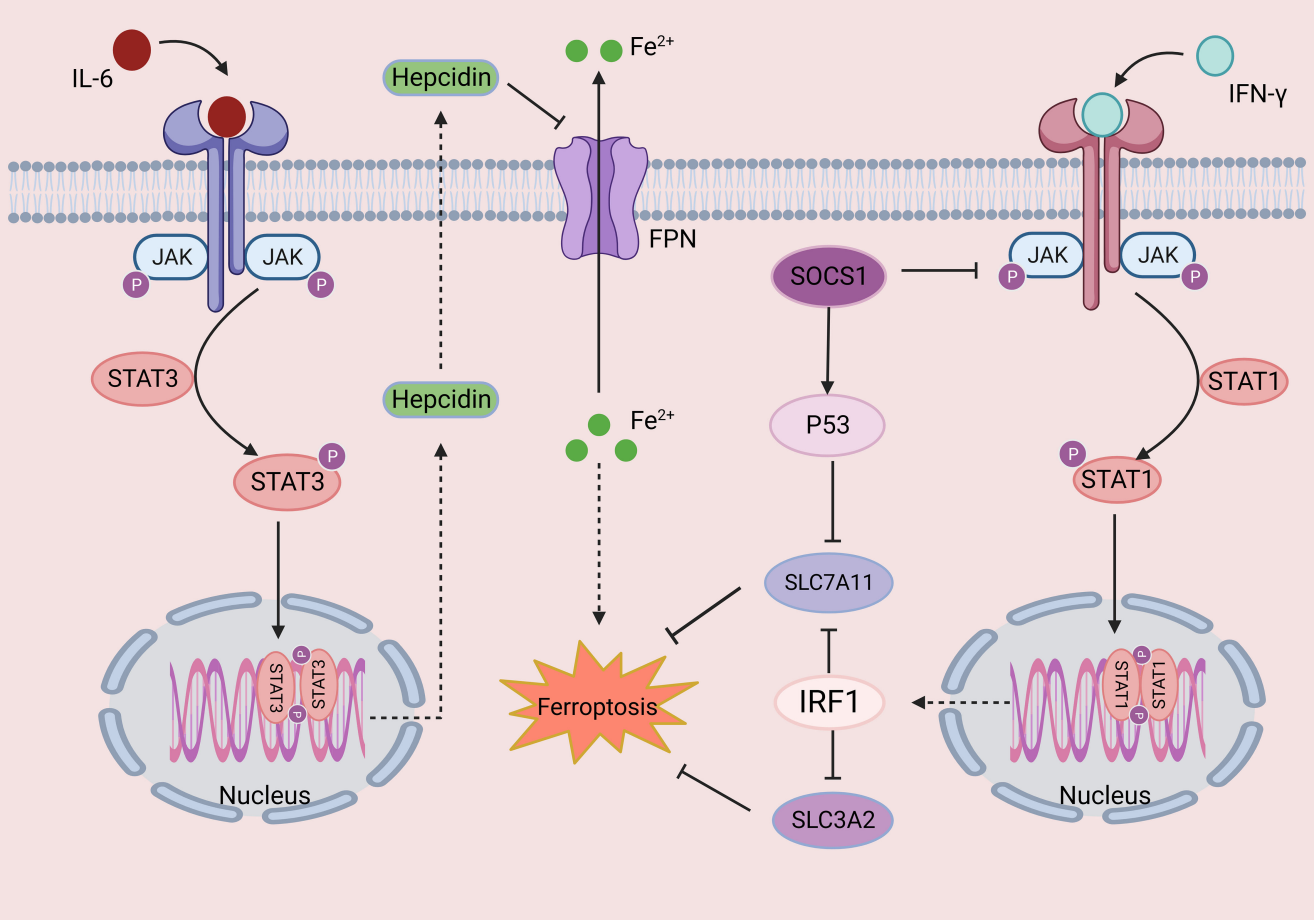
The role of the JAK-STAT signaling pathway in ferroptosis. Cytokines such as IL-6 and IFN-γ bind to their corresponding receptors, leading to oligomerization of the receptor, inducing JAK phosphorylation and activation. Activated JAKs mediate the phosphorylation of STATs and induce their dimerization. Finally, dimer translocation to specific DNA regulatory sequences regulates the transcription of target genes. Changes in iron homeostasis play an important role in ferroptosis. Hepcidin is an important mediator of iron homeostasis, and phosphorylated STAT3 can increase the expression of hepcidin, inhibiting iron export and resulting in ferroptosis. In addition, activated STAT1 inhibits two subunits of system X_c_^-^, SLC3A2 and SLC7A11, thereby leading to ferroptosis. This figure was created with BioRender.

The Janus kinase/signal transduction and transcriptional activation factor (JAK/STAT) pathway (153) is critical for initiating innate immunity, coordinating adaptive immune mechanisms, and ultimately suppressing inflammatory and immune responses (154, 155). Cytokines, such as TNF and IL-6, and their corresponding receptors and receptor oligomerization, facilitate JAK phosphorylation and activation upon receptor binding, thereby mediating STAT phosphorylation. Phosphorylated STAT dimerizes with other STAT family members with conserved SH2 domains. The dimer then translocates into the nucleus and binds to specific regulatory regions of DNA sequences to activate or repress target genes transcription (154, 156) (**Figure 3**). Negative regulators of JAK/STAT signaling include cytokine signaling (SOCS), activated STAT protein (PIAS), and protein tyrosine phosphatase (PTP) inhibitors (153).

Changes in iron homeostasis play an important role in ferroptosis (50). Hepcidin, an important factor is maintaining iron homeostasis, regulates systemic iron homeostasis by interacting with ferroportin. Hepcidin expression is affected by many factors, including serum and liver iron stores, erythropoiesis, hypoxia, inflammation and infection (157). Chronic inflammation occurs through the IL-6-JAK2-STAT3 signaling pathway and increases intracellular iron expression through microbial iron deprivation (157). Recent studies have revealed that auranofin, an anti-rheumatoid arthritis medication, induces hepcidin expression in human hepatocytes and mice via the JAK2-STAT3 signaling pathway (158). Similarly, dandelion polysaccharide inhibited the JAK-STAT signaling pathway and reduced iron element expression in a murine model, reducing the iron load and hepatocellular carcinoma (HCC) process (159).

The JAK/STAT signaling pathway also regulates ferroptosis-related molecules; for example, propofol, a widely used anesthetic, inhibits STAT3 expression by upregulating miR-125b-5p and accelerating ferroptosis in gastric cancer cells. Additionally, propofol was found to decrease GPX4 and SLC7A11 protein levels by suppressing STAT3 expression, decelerating gastric cancer growth *in vivo* (160). Similarly, propofol has been shown to enhance intracellular iron content and ROS levels by inhibiting STAT3 expression, exacerbating ferroptosis in colorectal cancer cells. Propofol also alters the expression of proteins related to ferroptosis in CRC cells, upregulating CHAC1 and PTGS2 expression and inhibiting GPX4 expression (161). Artesunate induces apoptosis, autophagy, and ferroptosis in diffuse large B-cell lymphoma (DLBCL) cells by impairing STAT3 signaling. IFNγ released by CD8+ T cells or natural killer cells plays a crucial role in antitumor host immunity (162). IFNγ activates the JAK/STAT pathway and downregulates SLC3A2 and SLC7A11, subunits of system X_c_^-^, in HCC cell lines. Additionally, IFNγ treatment enhances glutathione consumption, induces G0/G1 phase cell cycle arrest, increases lipid peroxidation, and sensitizes cells to ferroptosis activators (163). Quantitative PCR (qPCR) and STAT1 chromatin immunoprecipitation (ChIP) assays showed that IFNγ treatment enhances the binding of STAT1 to the SLC7A11 transcription start site (TSS) *in vivo*. STAT1 deficiency reversed the effects of IFNγ on SLC7A11 downregulation, IRF1 upregulation, lipid peroxidation induction, and erastin- or RSL3-induced cell death (164). In addition to causing tumors, IFNγ also inhibits GSH synthesis via the JAK1-2-STAT1-SLC7A11 pathway and triggers ferroptosis in retinal pigment epithelial cells, ultimately leading to macular degeneration *in vivo* (165).

Previously, we highlighted the role of the SOCS family in exerting negative feedback regulation on the JAK-STAT pathway, preventing sustained activation, in addition to its recently discovered association with ferroptosis (166). SOCS1 sensitizes cells to ferroptosis by regulating p53 target gene expression, through reducing SLC7A11 and GSH levels (167). In addition, a simulated peptide targeting the SOCS protein controlled the JAK/STAT signaling pathway and demonstrated anti-inflammatory, antioxidant, and renal protective effects in a T2DM mouse model (168).

### 3.3 Inflammasome signaling pathway in ferroptosis

**Figure 4:**
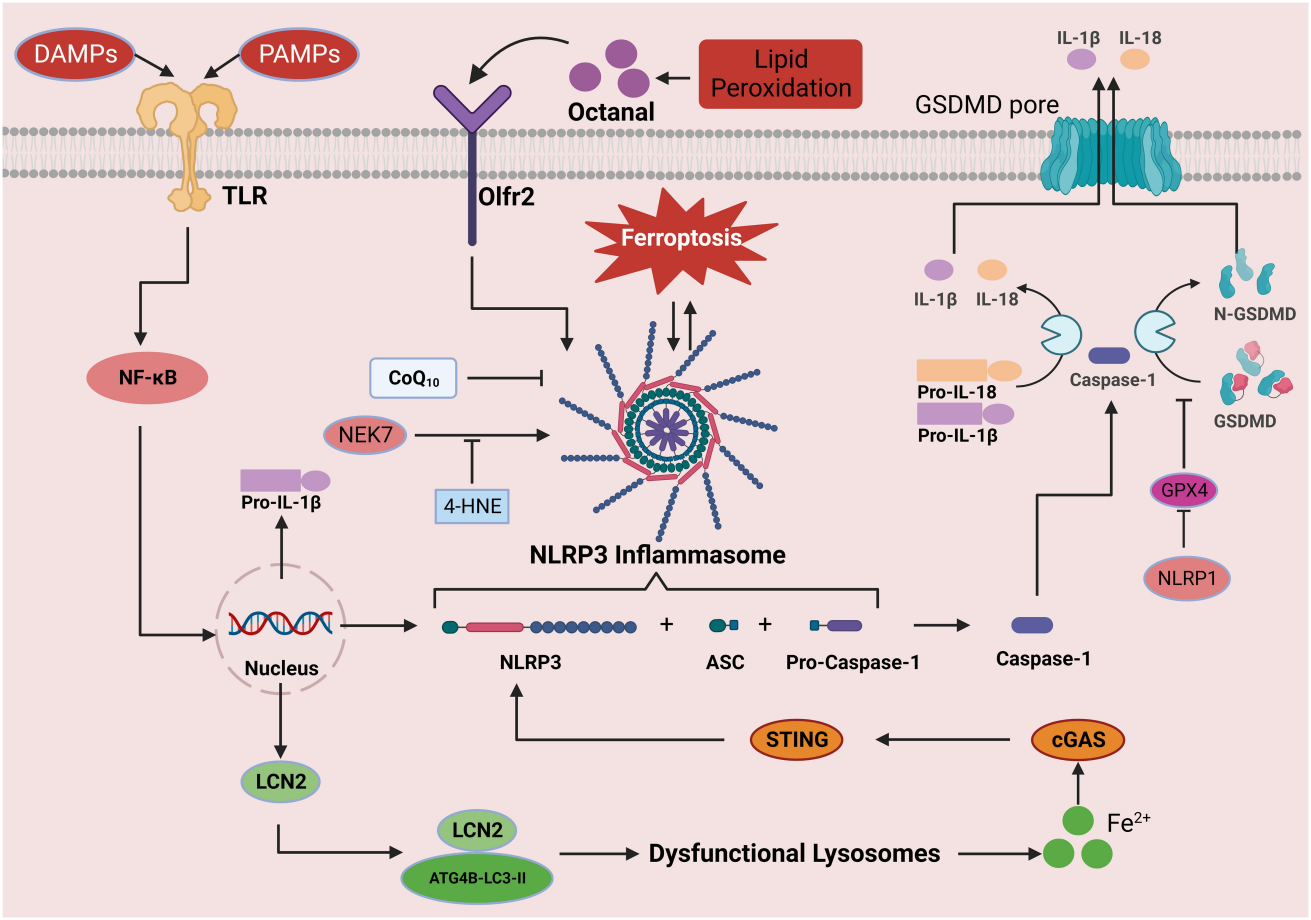
The role of the inflammasome signaling pathway in ferroptosis. Activation of the NLRP3 inflammasome can be divided into the priming and effector processes. The priming process includes the recognition of PAMPs/DAMPs by TLRs, which induces the expression of NLRP3, pro-IL-1β, and pro-IL-18 proteins through NF-κB signal transduction. Oligomerization and activation of the NLRP3 inflammasome results in the conversion of pro-caspase-1 to its active form, caspase-1. Caspase-1 shears the downstream molecules pro-IL-1β, pro-IL-18, and GSDMD, leading to the release of IL-1β and IL-18 and the formation of N-GSDMD. N-GSDMD enters the cell membrane, forms a pore structure, releases mature IL-1β and IL-18 into the extracellular space, induces pyroptosis, and causes inflammation. In this process, iron drives NLRP3 inflammasome formation through the cGAS-STING pathway, while GPX4 blocks GSDMD cleavage and inhibits the inflammasome pathway. In addition, octanal-induced lipid peroxidation contributes to NLRP3 inflammasome production, but 4-HNE binds to NLRP3, thereby hindering its interaction with NEK7 and inhibiting inflammasome activation. This figure was created with BioRender.

Inflammasomes are intracellular protein complexes that coordinate defense mechanisms against infections and physiological abnormalities. The classical inflammasome is comprises a sensor protein (mainly NLR family proteins and PYHIN family proteins), an adaptor protein (ASC), and pro-caspase-1 (caspase effector) through the pyrin domain (PYD) and/or caspase raise structure domain (CARD) that combines to form polymer protein complexes (169). The four main members of the inflammasome family are NOD-like receptor protein 1 (NLRP1), NOD-like receptor protein 3 (NLRP3), NOD-like receptor C4 (NLRC4), and absent in melanoma 2 (AIM2) (170). The NLRP3 inflammasome has been well studied and is known to be activated by various pathogens and molecular patterns released during cell injury or in response to environmental stimuli (171). After NLRP3 activation, ASC and pro-caspase-1 assemble the NLRP3 inflammasome, which mediates the self-cleavage of pro-caspase-1 and formation of the active caspase-1 p10/p20 tetramer. Activated caspase-1 further converts pro-IL-18 and pro-IL-1β to their mature forms. Active caspase-1 clears the porin Gasdermin D (GSDMD), releasing its N-terminal domain to the plasma membrane, which subsequently forms a pore that causes cell lysis (pyroptosis) and mediates the release of mature IL-18 and IL-1β, enhancing the inflammatory response (172) (**Figure 4**). Furthermore, studies have demonstrated that deubiquitinases (DUBs) exert regulatory control over the activity of caspase-1 and govern IL-1β secretion by orchestrating inflammasome assembly (173, 174). In addition, NLRP3 inflammasome-driven inflammation relies on cellular stress and dysfunction (175). NLRP3 activation stimulates specific pressures on organelles, leading to organelle stress and dysfunction (such as endoplasmic reticulum stress, mitochondrial ROS production, and mtDNA release); these processes directly influence NLRP3 through a series of specific PTMs, coordinating NLRP3 inflammasome activation and subsequent NLRP3-driven inflammation (176). As mentioned earlier, organelles, such as the mitochondria and ER, play a crucial role in lipid peroxidation during ferroptosis, suggesting that organelles may serve as platforms and sensors for regulating both the NLRP3 inflammasome and ferroptosis.

The NLRP3 inflammasome is one of the most studied and well-understood multiprotein complexes and has been linked to ferroptosis. In a dry age-related macular degeneration (AMD) mouse model, the level of lipid carrier protein 2 (LCN2) in retinal pigment epithelium (RPE) cells increased, and in addition to maintaining the steady state of iron, regulated macroautophagy/autophagy. LCN2 forms a complex with ATG4B, known as the LCN2-Atg4B-LC3-II complex, which plays a regulatory role in ATG4B activity and LC3-II lipidation, thereby impeding autophagosome maturation. Subsequently, excessive intracellular iron accumulation triggers NLRP3 inflammasome activation through the cGAS-STING1 pathway and further induces lipid peroxidation, oxidative stress, and ferroptosis (177). Ferroptosis was observed in pulmonary artery endothelial cells (PAECs) and lung tissues of pulmonary hypertension rats induced by monocrotaline (MCT), characterized by reduced cell viability, increased LIP and lipid peroxidation, upregulated NOX4 expression, and downregulated GPX4 and FTH1 expression. Further experiments demonstrated that ferroptosis-induced inflammation depends on HMGB1/TLR4 signaling activation, which activates the NLRP3 inflammasome *in vivo*. It has been proposed that pulmonary arterial endothelial cell ferroptosis mediates the progression of pulmonary hypertension through the MCT-induced HMGB1/TLR4/NLRP3 inflammasome signaling pathway in rats (178). In other studies using MCT to establish a rat model of pulmonary hypertension (PH), peroxiredoxin 6 (PRDX6) was found to regulate ferroptosis in PAECs through HMGB1 release and activation of the TLR4/NLRP3 inflammasome signaling pathway (179). In a study investigating the potential pathogenic role of ferroptosis in imidacloprid (IMI)-induced renal injury *in vivo*, ferroptosis inhibition with ferrostatin (Fer-1) blocked IMI-induced NLRP3 inflammasome activation (180). C57BL/6J and NLRP3 mice were used to establish a middle cerebral artery occlusion (MCAO) model to investigate the effect of NLRP3 deficiency on ferroptosis after cerebral ischemia-reperfusion injury (CIRI) *in vitro* and *in vivo*. Inhibition of the NLRP3 inflammasome alleviated CIRI by suppressing ferroptosis and inflammation (181). Other inflammasomes are also activated during ferroptosis. Excessive ferroptosis and inflammasome activation have been observed in rat oxidative stress and placental trophoblast cell models. Silencing the NLRP1 inflammasome increases GPX4 and GSH expression, thereby reducing intracellular MDA accumulation and cell death. However, the opposite phenomenon is observed when NLRP1 activators are applied (182). In a further study of the mechanism of ferroptosis on inflammasomes, after applying Fer-1 and erastin, the expression of NLRP3, NLRP1, IL-1β, and caspase-1 increased, indicating a mutually restrictive relationship between ferroptosis and the NLRP1 inflammasome (182).

Ferroptosis is an iron dependent, lipid peroxidation-driven mode of programmed cell death. Impaired clearance or excessive production of lipid peroxides can lead to their accumulation, reaching lethal levels and triggering ferroptosis. Recent studies have identified a potential association between lipid peroxidation and inflammasome activation. For example, octanal is present in mouse and human plasma and increases with a high-fat diet. Octanal is produced by lipid peroxidation. The octanal receptor Olfr2 in mice and OR6A2 in human vascular macrophages bind to TLR4 and induce inflammasome activation in response to octanal, leading to the production and secretion of IL-1α and IL-1β proteins (183). In contrast, coenzyme Q10 supplementation enhances mitochondrial function while reducing the production of reactive oxygen species and lipid peroxides, ultimately hindering NLRP3 inflammasome formation and cell death (184). The above studies show that lipid peroxidation promotes inflammasome formation. However, some studies have shown that lipid peroxidation inhibits inflammatory corpuscle formation. Hsu et al. showed in mouse models of acute lung injury and sepsis that treatment with 4-HNE, a lipid peroxidation product, or increasing endogenous 4-HNE by inhibiting GPX4, reduced inflammasome activation. Mechanistically, 4-HNE independently inhibits NLRP3 inflammasome activation without involving NF-κB signaling and Nrf2, while it has no impact on NLRC4 or AIM2 inflammasomes. Additionally, 4-HNE directly binds to NLRP3 and hinders its interaction with NEK7 (185). Therefore, further studies are needed to determine the impact of lipid peroxidation on inflammasome activation and ferroptosis.

### 3.4 NF-κB signaling pathway in ferroptosis

**Figure 5:**
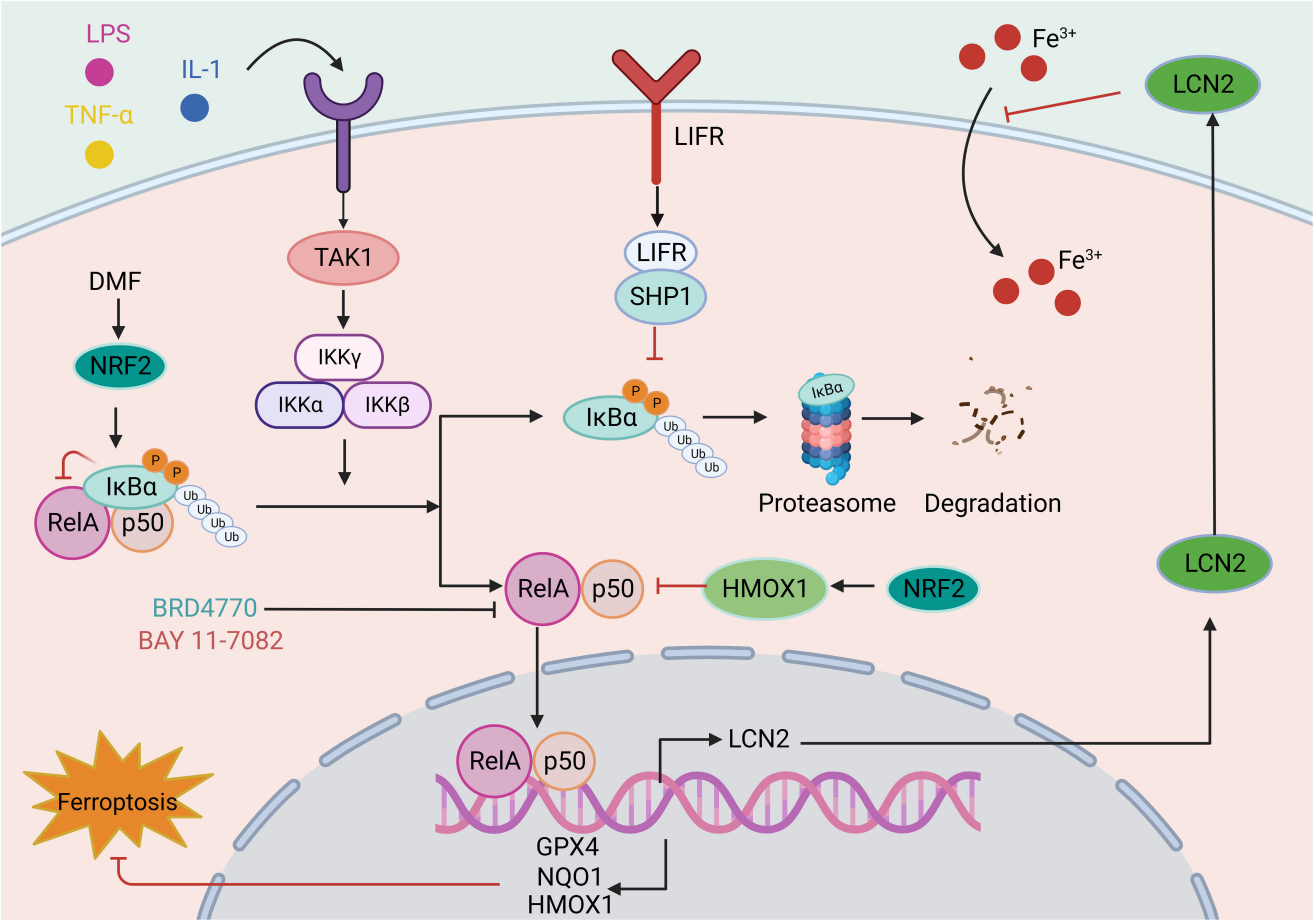
The role of the NF-κB signaling pathway in ferroptosis. The classical NF-κB pathway is triggered by signals from various immune receptors, such as LPS, TNF, and IL-1. Under resting conditions, IκBα binds to NF-κB dimers and sequesters NF-κB activity. These immune molecules and receptors activate TAK1, which phosphorylates IKK beta, activating IKK compounds. Subsequent IKK complexes mediate the phosphorylation and subsequent degradation of IκBα. The released NF-κB dimer is then transferred to the nucleus and regulates target gene transcription. The involvement of the NF-κB in ferroptosis is mainly reflected in the reduced transcription of antioxidant molecules, such as GPX4, NQO1, and HMOX1, indicating the role of the NF-κB pathway in oxidative stress. For example, LIFR depletion enhances the ubiquitination and degradation of IκBα and positively regulates NF-κB activation, further promoting the secretion of LCN2, which sequesters extracellular iron. In addition, drugs such as BRD4770 and BAY 11-7082 exert antiferroptotic effects by inhibiting the NF-κB pathway. This figure was created with BioRender.

Nuclear factor-κB (NF-κB) is an important nuclear transcription factor involved in the inflammatory response and immunity and can regulate cell apoptosis and the stress response (186). The NF-κB family comprises five members: RelB, RelA/p65, p50 (NF-κB1), p52 (NF-κB2), and c-Rel. These proteins can form various heterodimers or homodimers (such as the common p50/RelA heterodimer) and bind specifically to the promoter κB site, regulating target gene expression. NF-κB activation occurs through the classical and nonclassical NF-κB signaling pathways, both of which have different activation mechanisms (187). The classical NF-κB pathway is activated within minutes after exposure to proinflammatory signals, and involves TGFβ- associated kinase 1 (TAK1)-mediated activation of the IKK complex (consisting of catalytic (IKKα and IKKβ) and regulatory (IKKγ) subunits), which mediates the phosphorylation and subsequent degradation of IκBα (an IκB family protein), leading to the nuclear translocation of the NF-κB heterodimer RelA/p50. In addition, NF-κB binds to specific DNA elements to regulate the transcription of target genes (188) (**Figure 5**).

Existing studies have demonstrated that ferroptosis is involved in the NF-κB signaling pathway. In investigations on the neuroprotective effects of Icariside II (ICS II) preconditioning in mice with focal cerebral ischemia induced by transient MCAO and primary astrocytes challenged with oxygen-glucose deprivation (OGD), ICS II preconditioning can protect astrocytes from lethal OGD exposure by promoting Nrf2 nuclear translocation and activating the OXPHOS/NF-κB/ferroptosis axis (189). Dimethyl fumarate (DMF) is an FDA-approved treatment for multiple sclerosis (MS) and plays a protective role in various neurological diseases. DMF activates Nrf2, leading to the upregulation of the key ferroptosis factors HMOX1, NQO1, and GPX4 by modulating IκBα and inhibiting NF-κB signaling pathway activation. This mechanism ultimately protects cells from oxidative stress and ferroptosis (190). Leukemia inhibitory factor receptor (LIFR) expression is reduced in hepatocellular carcinoma, causing activation of NF-κB signaling through SHP1 and upregulation of the iron-chelating cytokine LCN2. This process inhibits extracellular iron import and reduces cellular sensitivity to ferroptosis inducers (191). In another study, aspirin was found to trigger ferroptosis in HCC cells by restricting NF-κB p65-activated SLC7A11 transcription (192).

Nonclassical NF-κB activation is also involved in ferroptosis. NIK is a serine/threonine kinase that mediates the activation of the noncanonical NF-κB2 pathway and is abnormally activated in numerous liver diseases. NIK phosphorylates and activates the NF-κB kinase subunits of alpha (IKK alpha) inhibitors, and the liver NIK/IKK alpha cascade inhibits liver regeneration. Liver cell-specific ablation of NIK or IKK alpha can prevent lipid peroxidation in liver cells and alleviate acetaminophen (APAP)-mediated hepatotoxicity in mouse liver tissue and mortality (193). Overall, the above findings indicate that targeting NF-κB signaling is a promising approach for intervening in ferroptosis and has therapeutic effects on various diseases.

NF-κB signaling pathway interventions involving ferroptosis activators or inhibitors have been investigated in *in vitro* and *in vivo* models. The well-characterized ferroptosis activator RSL3 inhibits GPX4 activity, resulting in lipid peroxidation (94). The NF-κB pathway has been investigated in RSL3-induced ferroptosis in glioblastoma cells, in which it activated the NF-κB pathway, increased lipid ROS levels, and decreased the expression of ferroptosis-related proteins (GPX4, ATF4, and SLC7A11/xCT). Furthermore, inhibition of NF-κB signaling with BAY 11-7082 alleviated ferroptosis and attenuated the antitumor effect of RSL3 *in vivo* (194). Similarly, a histone methyltransferase inhibitor (BRD4770) was used as a novel inhibitor of ferroptosis in smooth muscle cells (SMCs) induced by cystine deprivation, imidazolone erastin or RSL3.The activation of NF-κB signaling induced by these three stimuli was reversed by BRD4770, protecting SMCs from ferroptosis (195). Studies on the effect and mechanism of cigarette tar on the progression of atherosclerosis (AS) have demonstrated that cigarette tar aggravates atherogenesis by promoting lipid peroxidation and ferroptosis in macrophages. The use of ferroptosis inhibitors (FER-1 and DFO), hepcidin knockdown, or SLC7A11 overexpression reversed the aforementioned changes. Additionally, inhibition of NF-κB with BAY11-7082 reversed tar’s regulatory effect on the hepcidin/FPN/SLC7A11 axis, thereby inhibiting macrophage ferroptosis. It has been confirmed that cigarette tar induces macrophage ferroptosis through the NF-κB-activated hepcidin/FPN/SLC7A11 pathway, thereby promoting the progression of atherosclerosis (196). The findings presented herein provide compelling evidence that NF-κB signaling pathway activation is indispensable for ferroptosis. In addition, in a rat model of lipopolysaccharide (LPS)-induced cardiac dysfunction, LPS increased iron accumulation in the myocardium, downregulated the expression of iron transporters (FPN, SLC40A1) and transferrin receptor (TfR), and upregulated the expression of FTL and FTH1. LPS also increased lipid peroxidation in rat hearts by reducing GPX4 expression, while the ferroptosis inhibitor Fer-1 alleviated the adverse effects caused by LPS and improved LPS-induced cardiac dysfunction by inhibiting the TLR4/NF-κB signaling pathway (197).

### 3.5 MAPK signaling pathway in ferroptosis

**Figure 6:**
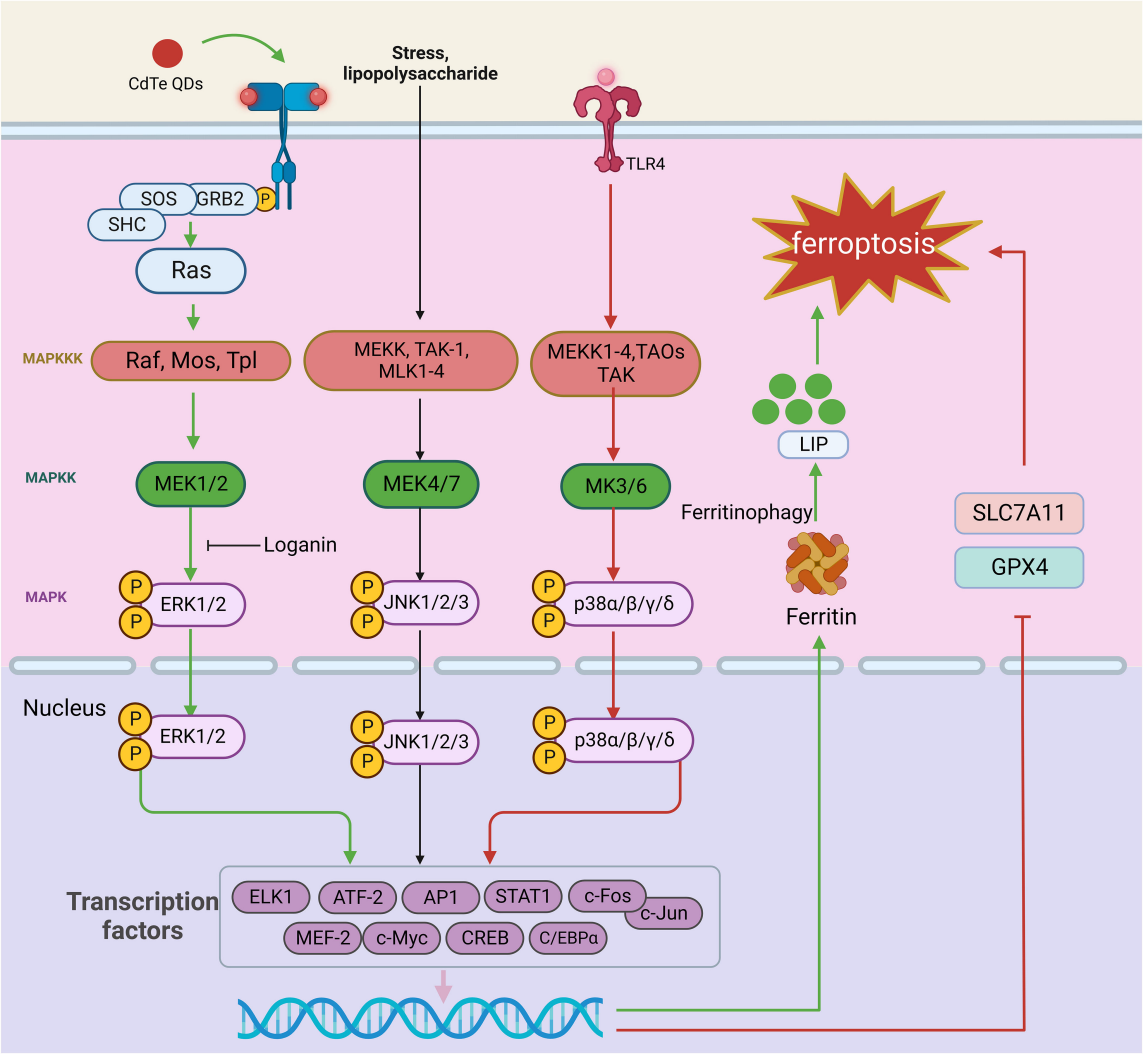
The role of the MAPK signaling pathway in ferroptosis. The MAPK signaling pathway is a cascade of phosphorylation involving MAPKKK, MAPKK, and MAPK. The representative MAPK signaling pathways include the ERK, JNK, and p38MAPK signaling pathways. When the ERK signaling pathway is activated, the external stimulus binds to the corresponding receptor on the cell membrane, which causes the receptor to form a dimer, and this dimerization activates its associated tyrosine kinase. The tyrosine phosphorylated on the receptor binds to the SH2 domain of Grb2 located on the cell membrane, which binds to SOS and activates Ras. Ras family activation can further activate MAPKK kinases. Activation of MAPKK kinase activates MEK1/2, which subsequently recognizes and phosphorylates ERK1/2. External stimuli, inflammatory factors, physical and chemical stimuli first activate MEK1-4, TAOs, TAK, and ASK in the MAPKK kinase family; these proteins mediate the p38 pathway and gradually activate MEK3/6 and p38 isoforms. In the JNK signaling pathway, multiple signals are mediated by factors that phosphorylate MEKK, MLK1-4, and TAK-1 and activate MAPKK kinases, which continue to activate MEK4/7, followed by JNK1/2/3. Finally, activated MAPK translocates to the nucleus, phosphorylates certain transcription factors (such as AP-1), promotes the release of cytokines, and participates in cellular regulation. TLR4-p38 MAPK pathway activation reduces SLC7A11 and GPX4 expression, leading to ferroptosis. CdTe QDs decrease NRF2 and ERK1/2 phosphorylation, activating ferritin autophagy and triggering ferroptosis. In addition, loganin attenuates ferroptosis by inhibiting ERK1/2 activation *in vivo*. This figure was created with BioRender.

Silk cleaves the original activated protein kinase (MAPK) family of mammals, including extracellular signal regulating kinase (ERK), c-Jun NH2-terminal kinase (JNK), and p38 lightning. The MAPK pathways are activated by a range of external and internal stimuli, such as growth factors, cytokines, hormones, and cellular stressors, including oxidative and ER stress. These pathways regulate various cellular activities, including proliferation, differentiation, survival, and apoptosis. The classical MAPK signaling pathway is composed of a tertiary conserved kinase pattern, MAPKK kinase (MAPKKK), MAPK kinase (MAPKK), and MAPK, which transfer the cascade-amplified signal into the nucleus and regulate numerous important physiological and pathological cellular processes at the gene level. The representative MAPK signaling pathways include the ERK1/2, JNK, and p38MAPK signaling pathways (198) (**Figure 6**).

The ERK signaling pathway is often activated by biologically active substances such as hormones and neurotransmitters. Stimulation with the membrane receptor, containing the Src structure of the homologous region 2 domain (Src homology 2 SH2) or phosphorylated tyrosine combination structure domain (phosphotyrosine-binding domains, PTB) proteins [such as growth factor receptor-bound protein 2 (Grb2), and intracellular exchange factors (SOS S)], results in a series of changes that activate the Ras family. Ras family activation can further activate MAPKK kinases. The MAPKK kinase family members identified to date include Raf, Mos, and Tpl. Activation of MAPKK kinase in turn activates MAPK kinase (MEK1/2), which subsequently recognizes and phosphorylates ERK1/2, which contains a two-site threonine (T) and tyrosine (Y). Activated ERK1/2 then continues to react with substrates in the cytoplasm and enters the nucleus, where it mediates the transformation of transcription factors. Thus, activated ERK1/2 plays an important regulatory role in physiological and pathological processes at the gene level (199).

There are four subtypes of P38, α, β, γ, and δ, which can be activated by external stimuli, inflammatory factors, and physical and chemical stimuli. These stimuli first activate MEKK1-4, TAOs, transforming growth factor B activating kinase (TAK) and ASK, which mediate the p38 pathway and gradually activate MAPK kinases (MEK3/6) and p38 isoforms. Similar to ERK1/2, p38 can affect the expression of some transcription factors, thus exerting regulatory effects (200).

In the JNK signaling pathway, cytokines, physical and chemical stimuli, stress, and other signals are mediated by factors that phosphorylate MEKK, MLK-2, MLK-3, MLK-12, MLK-13, and transforming growth factor B activated kinase 1 (TAK-1) and activate MAPKK kinase to activate MEK4/7. Subsequently, JNK isoforms (JNK1/2/3) are activated. Finally, activated JNK translocates to the nucleus and phosphorylates certain transcription factors (such as AP-1), promoting the release of cytokines and contributing to cellular regulation (200).

MAPK signaling pathway activation is known to be intricately associated with ferroptosis. Yang et al. demonstrated that cetuximab, which is approved for treating RAS wild-type metastatic colorectal cancer (mCRC), enhances the cytotoxic effect of RSL3 on KRAS-mutant CRC cells. p38 MAPK activation potentiates RSL3-induced ferroptosis by inhibiting the Nrf2/HO-1 axis (201). In a neonatal rat hypoxia-ischemia (HI) model, TLR4-p38 MAPK pathway activation induces the production of the proinflammatory cytokines IL-18, IL-6, and IL-1β, significantly upregulates TLR4 expression, increases p53 levels, decreases SLC7A11 and GPX4 levels, and causes mitochondrial damage, inducing ferroptosis in hippocampal neurons. TAK-242, a specific TLR4 antagonist, can inhibit the expression of ferroptosis-related proteins, reduce the expression of ferroptosis-related genes and the proinflammatory environment, alleviate oxidative stress and mitochondrial damage, and ultimately improve the activation of ferroptosis in hippocampal neurons after HIBD (202). Similarly, OGD induces ferroptosis in neuronal cells by activating the TLR4-p38 MAPK pathway and increasing MDA accumulation. However, inhibition of p38 with SB203580 alleviates OGD-induced ferroptosis by upregulating SLC7A11 and GPX4 expression in neuronal cells (202). ERK also mediates inflammatory responses and ferroptosis. LCN2 is associated with ARDS, and silencing it prevents lung injury by inhibiting ferroptosis-mediated lung inflammation and oxidative stress through MAPK/ERK pathway inhibition (203). Cadmium telluride quantum dots (CdTe QDs) decrease NRF2 expression, ERK1/2 phosphorylation, and, subsequently, ferritin autophagy activation. The degradation of ferritin heavy chain 1 (FTH1) in lysosomes and proteasomes releases free iron ions, leading to ferroptosis in macrophages (204). Notably, several drugs targeting the ERK pathway have been shown to have desirable therapeutic effects in alleviating ferroptosis. Loganin, a major iridoid glycoside compound isolated from *Corni fructus*, has been used as an anti-inflammatory agent in various pathological models. Loganin inhibits ERK1/2 activation in cisplatin-induced acute kidney injury induced by cisplatin, attenuating renal cell ferroptosis and promoting the release of the proinflammatory cytokines TNF-α, IL-6, and IL-1β (205). In summary, inhibiting MAPK signaling with targeted therapeutics may be effective in alleviating ferroptosis and related diseases; however, specific drugs with this capability have yet to be discovered.

Typical features of ferroptosis, such as iron overload and excessive lipid peroxidation, have been shown to affect MAPK pathway activity. One study demonstrated that iron excess upregulated p38 and c-FOS phosphorylation, leading to aggravated hepatocyte injury and increased lipid peroxidation. *In vivo*, taxifolin, a natural compound, reverses iron-mediated oxidative stress, enhances REDOX status, and prolongs hepatocyte survival by inhibiting MAPK signaling pathway activation (206). However, iron overload in CTX-induced muscle injury inhibits ERK1/2 and p38 phosphorylation, increasing oxidative stress and impairing muscle regeneration. GDC-0879 functions as a Braf/MAPK-targeted inhibitor that prevents PUFA-mediated lipid peroxidation and reverses CoQ deficiency, thereby reversing podocyte injury and improving kidney disease *in vivo* (207). The findings of the aforementioned studies suggest that exploring the link between the MAPK signaling pathway and ferroptosis may reveal intervention targets for ferroptosis-related diseases.

As mentioned above, by exploring the relationship between inflammation-related signaling pathways and ferroptosis, it has been demonstrated that inflammation-related signaling pathways can participate in and regulate ferroptosis, and ferroptosis can also regulate inflammation and affect cell biological functions. These findings suggest a close relationship between ferroptosis and inflammation in both physiological and pathological functions. Targeting these pathways may effectively intervene in ferroptosis, leading to therapeutic effects in various disease models *in vivo*. Inflammation-related signaling pathways have also been implicated in other types of cell death, such as necroptosis and pyroptosis. Notably, unlike in other forms of cell death, the mechanism of ferroptosis is uniquely linked to inflammation (**Table 1**). Additionally, numerous studies have revealed the involvement of inflammation-related pathways in regulating iron metabolism, lipid peroxidation, and REDOX systems. These findings enhance our understanding of the mechanisms underlying these pathways in ferroptosis. Based on these findings, several drugs targeting inflammatory signaling pathways have been shown to impact iron metabolism and lipid peroxidation, leading to therapeutic outcomes that either promote or inhibit ferroptosis (**Table 2**).

**Table 1:** Summary of the similarities and differences in the relationship between each form of cell death and inflammation.

	Ferroptosis	Apoptosis	Necroptosis	Pyroptosis	Autophagy
**Definition**	A novel form of programmed cell death caused by iron-dependent lipid peroxidation and ROS overproduction	The autonomic and orderly death of cells, controlled by genes, to maintain homeostasis .	A regulated mode of necrotizing cell death mediated by RIP1 and RIP3 kinase	A form of regulatory cell death activated by inflammasomes .	A process involving engulfing cytoplasmic proteins or organelles and wrapping them into vesicles that fuse with lysosomes, forming autolysosomes that degrade the encapsulated contents
**Morphology features**	Mitochondria are decreased, membrane density is increased, and mitochondrial body cristae are decreased	The cells shrink, the membrane structure remains intact, the nucleus is condensed, the DNA in the nucleus is broken, and the cells are wrapped into vesicles that agglomerate to form apoptotic bodie	The organelles are swollen, the membranes are broken down, and the cytoplasm and nucleus are decomposed	The membrane is ruptured, the cells become swollen and expanded with bubble-like protrusions, the nuclei remain intact, and the DNA is fragmented	The Golgi apparatus, endoplasmic reticulum and other organelles become swollen, the cytoplasm is amorphous, the nucleus is fragmented and pyknotic, a large number of phagocytic vacuoles are formed, the cytoplasmic membrane becomes unspecialized, and cell membrane blebbing may occur
**Inducement**	Impairment of iron-dependent intracellular ROS metabolism	Hormone and growth factor imbalance, physical and chemical factors, and immune factors	Extracellular stimulation	Pathogen infection	Nutritional deficiency, abnormal energy metabolism, ischemia, hypoxia, and pathogen infection
**Molecular biomarkers**	HMOX1, PTGS2, GPX4, SLC7A11, FSP1, TFR1, NRF2, P53, ACSL4	Caspase-3, BCL-2, BAX, p53, FAS	RIP1, RIP3, MLKL	NLRP3, GSDMD, Caspase-1, IL-1β, IL-18	P62, ATG5, ATG7, Beclin-1, ULK1, LC3, FIP200, ATG13, ATG101, ATG14L, ATG16L1, ATG12
**Immune features**	Proinflammatory	Mostly anti-inflammatory	Mostly Proinflammatory	Proinflammatory	Mostly anti-inflammatory
**Major molecules related to inflammation**	SLC7A11, GPX4, FSP1	Caspase-3, Caspase-8, Caspase-9	RIP1, RIP3, MLKL	NLRP3, Caspase-1, GSDMD,	LC3, ATG5, ATG16L1, Beclin-1, ATG9L1, ULK1
**Major inflammatory signaling pathways**	JAK-STAT, NF-κB, inflammasome, cGAS-STING and MAPK signaling pathways	JAK-STAT, NF-κB, inflammasome, cGAS-STING and MAPK signaling pathways	JAK-STAT, NF-κB, inflammasome, cGAS-STING and MAPK signaling pathways	JAK-STAT, NF-κB, inflammasome, cGAS-STING and MAPK signaling pathways	JAK-STAT, NF-κB, inflammasome, cGAS-STING and MAPK signaling pathways

**Table 2:** Functions of drugs targeting inflammatory signaling pathways in ferroptosis-related diseases.

Inhibitors	Signaling pathways	Targets	Effects on ferroptosis	Functions	Experimental models	References
**Aspirin**	NF-κB	NF-κBp65	Aspirin triggers ferroptosis in hepatocellular carcinoma cells by restricting NF-κB p65-activated SLC7A11 transcription	Preventing the development of hepatocellular carcinoma (HCC)	HepG2 and Huh7 cells	(192)
**Cetuximab**	MAPK	p38 MAPK	Cetuximab promotes ferroptosis by enhancing the RSL3-induced increase in lipid ROS and MDA levels	Treating RAS wild-type metastatic colorectal cancer (mCRC)	Two KRAS mutant CRC cell lines, HCT116 and DLD-1	(201)
**ginsenoside Rd**	cGAS/STING	cGAS/STING	Significantly reduces GSH and GPX4 expression	Protects mice from CCl4-induced ALI	CCl4-induced ALI in C57BL/6 mice	(147)
**Lidocaine**	MAPK	p38 MAPK	Lidocaine reduces FTH1 and GPX4 induced by H/R significantly increases and H/R-induced Tf	Lidocaine attenuates hypoxia/reoxygenation-induced inflammation, apoptosis and ferroptosis in lung epithelial cells	Oxygen deprivation/reoxygenation-induced A549 cells	(208)
**propofol**	JAK-STAT	STAT3	The erastin-induced ferroptosis of gastric cancer cells is exacerbated by increasing intracellular ROS and Fe ^2+^ levels, the expression levels of GPX4 and SLC7A11 are inhibited	Inhibition of gastric cancer growth *in vivo*	SGC7901 and BGC823 cells after subcutaneous injection into the right flank of specific-pathogen-free male nude mice	(160)
			Propofol may enhance the iron level and ROS accumulation, upregulate the expression levels of CHAC1 and PTGS2, and downregulate the expression level of GPX4, including ferroptosis in colorectal cancer cells	Inhibition of colorectal cancer tumorigenesis	Human normal colonic epithelial NCM460 cells and human CRC SW480 cells	(161)
**propofol**	JAK-STAT	STAT3	Propofol regulates iron metabolism and prevents nerve cell damage caused by iron overload	To prevent the nerve cells of the damage caused by iron overload	Human SH-SY5Y cells	(209)
**artesunate**	JAK-STAT	STAT3	ART induces ferroptosis and exerts a synergistic effect with an inducer of ferroptosis in DLBCL cells	ART has antitumor effects on diffuse large B-cell lymphoma (DLBCL) cells	U2932, SU-DHL2, SU-DHL4, SU-DHL6, and 293 T cells	(162)
**dimethyl fumarate**	NF-κB	NF-κBp65	Dimethyl fumarate can inhibit ferroptosis of hippocampal neurons by upregulating the expression of HO-1, NQO1 and GPX4, thus improving cognitive impairment in CCH rats	Improve cognitive impairment in rats with CCH	CCH rat model	(190)
**loganin**	MAPK	ERK 1/2	Loganin treatment reversed the downregulation of GPX4 and upregulation of 4-HNE	Loganin could protect against ferroptosis in cisplatin-induced AKI	Cisplatin-induced AKI	(205)
**taxifolin**	MAPK	p38 MAPK	Reduced liver iron content and enhanced REDOX status	Taxifolin attenuates iron-induced liver cell injury and histopathological aberrations	Iron-treated rats	(206)
**alpha lipoic acid**	MAPK	p38 MAPK	Decreased MDA level and increased SOD activity	Ameliorates oxidative kidney injury induced by iron sucrose	Iron sucrose-induced oxidative kidney injury	(210)
**resveratrol**	MAPK	p38 MAPK	Reduces the production of lipid peroxides and increases the levels of antioxidants	Protects against damage caused by spinal cord ischemia	Spinal cord ischemia-reperfusion injury model in rats	(211)
**GDC-0879**	MAPK	ERK1/2	Upregulates GPX4 expression and prevents lipid peroxidation	Improvement of CoQ-deficiency kidney disease	Pdss2kd/kd mice Pdss2-depleted podocytes	(207)
**MGM**	MAPK/NF-κB	p-JNK	Upregulates MVA-mediated antioxidant capacity (Gpx4 and FSP1/CoQ10 axes) and impaired ACSL4-mediated renal lipid driver production	Improves DN induced by STZ in rats, demonstrating a renal protective effect	STZ-induced diabetic rat model	(212)

## 4 Ferroptosis, inflammation, and diabetic nephropathy

**Figure 7:**
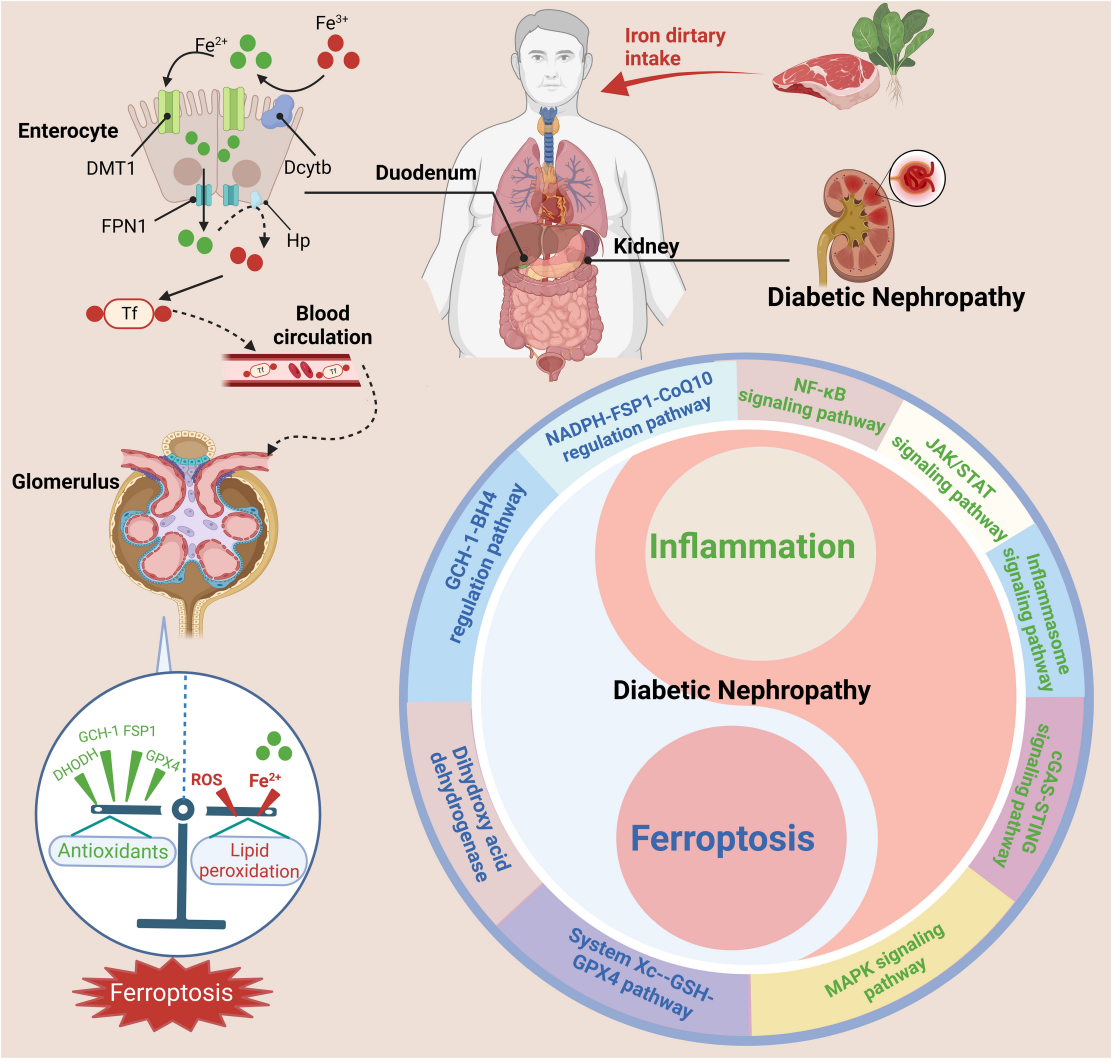
The interrelationship between ferroptosis and inflammation in DN. Nonheme iron (Fe^3+^) is mainly absorbed in the duodenum and upper jejunum. Trivalent iron is first reduced to divalent iron by DCYTB on the apical membrane of gastrointestinal mucosal cells and then enters the body through the apical membrane of intestinal cells through DMT1. After absorption in enterocytes, reduced iron Fe^2+^ is transported into the circulation by FPN1 and converted to Fe^3+^ by Hp. Fe^3+^ enters the blood and binds to Tf, after which it is delivered to the bone marrow or other iron-requiring tissues for utilization. The kidney also plays a key role in the maintenance of iron balance. The dysregulation of iron metabolism and imbalance of pro-inflammatory and anti-inflammatory cells can activate various inflammatory signaling pathways (such as the cGAS-STING, JAK-STAT, inflammasome, NF-κB, and MAPK pathways) and ferroptosis signaling pathways (such as the System X_c_^–^-GSH-GPX4 pathway, NADPH-FSP1-CoQ10 regulatory pathway, GCH-1-BH4 regulatory pathway, and dihydroxy acid dehydrogenase), exacerbating the progression of DN. This figure was created with BioRender.

DN is often considered to be the result of the interaction between hemodynamic and metabolic factors; however, its pathogenesis arises from a combination of multiple factors (11, 213–216). Previous studies have revealed inextricable associations among DN, inflammation, and ferroptosis. Recent studies have also shown regulating the occurrence and development of inflammation in DN can inhibit ferroptosis to a certain extent, alleviating DN progression (**Figure 7**).

Given the role of inflammation in ferroptosis, the regulation of inflammation-related pathways is beneficial in treating DN. HMGB1, a typical DAMP, is an endogenous mediator of inflammation that can be released through apoptosis, ferroptosis, and necrosis (217). Extracellular HMGB1 can induce signaling cascades that activate NF-κB, leading to the synthesis of proinflammatory cytokines. HMGB1 is activated in patients with DN and mesangial cells in response to high glucose levels. Additionally, HMGB1 may induce inflammation by activating the Toll-like receptor pathway in DN. Recently, HMGB1 was reported regulate ferroptosis through the RAS-JNK/p38 pathway and represents a potential therapeutic target in leukemia. HMGB1 levels are significantly increased in patients with DN, accompanied by dysregulation of ferroptosis-related molecules, including GPX4, PTGS2, NOX1 and ACSL4. In a further study, inhibiting HMGB1 levels in high glucose–exposed mesangial cells was found to restore cell proliferation, prevent ROS production, reverse ferroptosis, and reduce proinflammatory cytokine levels. These findings suggest that inhibiting HMGB1 has beneficial effects on glucose-induced ferroptosis, hyperoxidation, and mesangial cell inflammation. We investigated whether HMGB1 regulates Nrf2 signaling-mediated ferroptosis in DN. Both clinical DN samples and mesangial cells treated with high glucose exhibited reduced levels of Nrf2 and its downstream targets, including HO-1, NQO-1, GCLC, and GCLM. In addition, HMGB1 inhibition reversed glucose-induced TLR4/NF-κB activation and the reduction in Nrf2 and its targets in SV40 MES 13 cells, suggesting that HMGB1 is a novel regulator of ferroptosis via Nrf2. HMGB1 regulates glucose-induced activation of the TLR4/NF-κB signaling pathway in mesangial cells (218). “Multiomics” analysis combined with systemic pharmacology has been used to investigate the protective effect of mangiferin monosodium salt (MGM) on renal injury in STZ-induced DN rats with particular attention on kidney ferroptosis, inflammation, and podocyte insulin resistance (IR) signaling events. The study revealed excessive secretion of proinflammatory cytokines in the DN mouse kidney (IL-6, IL-18, TNF-α, and IL-1β) and severe renal interstitial inflammatory cell infiltration; MGM treatment protected against STZ-induced DN by reducing renal inflammation and podocyte injury caused by systemic insulin resistance. These effects are mainly attributable to the inhibition of the MAPK/NF-κB pathway and activation of the phosphorylated insulin receptor substrate 1 (Tyr608)/phosphorylated PI3K/phosphorylated Akt pathway in the DN rat kidney. MGM ameliorated renal ferroptosis in STZ-induced DN rats by upregulating mevalonate (MVA)-mediated antioxidant capacity (Gpx4 and FSP1/CoQ10 axis) and attenuating ACSL4-mediated renal lipid driver production (212).

In addition to modulating inflammation-related pathways to alleviate the occurrence of ferroptosis in DN, some studies have found that regulating ferroptosis can reduce inflammation, oxidative stress, and cell death in DN, thereby improving DN. For example, Shuquan Lv et al. found that San-Huang-Yi-Shen capsule (SHYS) significantly reduced the expression of inflammatory factors in the renal tissue of DN mice (219). In addition, SHYS reduced the elevated total iron concentration and FTH/FTL ratio in the renal tissues of these mice, indicating relief of the iron overload. Transferrin and STEAP3 levels were also decreased, indicating improved control of iron uptake by renal cells. Importantly, TEM results demonstrated that SHYS treatment significantly improved morphological abnormalities and mitochondrial disruption in the kidney cells of DN mice, indicating an improvement in ROS accumulation-triggered damage. Additionally, SHYS effectively increased the GSH/GSSG ratio and alleviated GSH depletion in the renal tissues of DN mice, providing sufficient GSH to scavenge lipid hydroperoxides. Moreover, SHYS significantly upregulated the protein levels of GPX4, SLC3A2, GCLC, and SLC7A11 associated with the cysteine/GSH/GPX4 axis in the kidneys of DN mice, promoting the expression of the GPX4 antioxidant system. However, inhibition of GPX4 abolished the protective effects of SHYS against inflammation, oxidative stress, and cell death. These findings suggest that by mitigating ferroptosis through reducing iron overload and activating the cystine/GSH/GPX4 axis pathway, SHYS may alleviate inflammation, oxidative stress, and cell death in DN (219).

Some anti-inflammatory drugs can slow the progression of DN by inhibiting ferroptosis. For example, platycodin-D (PD) has anti-inflammatory activity (220). It can inhibit HG-induced ferroptosis in HK-2 cells by upregulating GPX4 expression (221). Quercetin (QCT) is a flavonoid found in fruits and vegetables with various pharmacological effects, including antioxidative stress, anti-ferroptosis, and anti-inflammatory activities (222). Nrf2 can directly control the expression of the HO-1 enzyme encoded by the HMOX1 gene. The critical role of Nrf2-mediated HO-1 expression in anti-inflammatory activity has been demonstrated in a series of *in vitro* and *in vivo* experiments (223, 224). Some studies have shown that QCT can alleviate ROS-mediated mitochondrial damage and inflammation through the Nrf2/HO-1 signaling pathway. Feng et al. demonstrated that QCT effectively reduces intracellular iron concentration and lipid peroxidation by activating the Nrf2/HO-1 signaling pathway. Moreover, a Nrf2 gene complementation assay revealed that the Nrf2 inhibitor ML385 induced ferroptosis and diminished the renoprotective effect of QCT on DN, further highlighting the crucial role of Nrf2/HO-1 signaling in the effect of QCT for treating DN progression. Therefore, both *in vitro* and *in vivo* studies suggest that QCT is a promising candidate for treating DN due to its ability to target the Nrf2/HO-1 signaling pathway and dose-dependently inhibit ferroptosis in renal tubular epithelial cells during DN (225). Calycosin, an isoflavone, has immunomodulatory, anti-inflammatory, antiviral, and antioxidant properties (226). Huang D et al. found that calycosin reduced free iron import and lipid ROS in HK-2 cells, inhibiting HG-induced ferroptosis (227).

Studies on ferroptosis-related proteins and targets *in vivo* have demonstrated that regulating these proteins and targets can significantly improve inflammation and ferroptosis in DN. For example, a study was conducted on the role of peroxide reduction protein 6 (Prdx6) in the HG-mediated induction of podocyte damage. Prdx6 overexpression increased cell viability and inhibited HG-induced podocyte death, inflammation, and destruction in MPC5 cells. Prdx6 overexpression also suppressed HG-induced ROS and MDA production while restoring SOD and GSH activities in MPC5 cells. These results suggest that Prdx6 overexpression can inhibit HG-induced oxidative stress injury. Prdx6 overexpression also abolished HG-induced ferroptosis, as reflected in the inhibition of iron accumulation and increased expression of SLC7A11 and GPX4. Ferroptosis activator erastin abolished the protective effect of Prdx6 on HG-induced podocyte injury. The Sp1 protein directly regulates Prdx6 transcription in podocytes by binding to three Sp1-responsive elements in the Prdx6 promoter. However, silencing Sp1 eliminates its influence. Overall, the upregulation of Prdx6 expression mediated by Sp1 protects against podocyte injury in DN by reducing inflammation, oxidative stress, and ferroptosis (228). However, Li Q et al. found that the expression level of circular ASAP2 was downregulated and the expression level of miR-770-5p was upregulated in DN *in vitro* and *in vivo* models (229). In addition, circ ASAP2 reduced inflammation and ferroptosis in DN through the miR-777-5p/SOX2/SLC7A11 axis (229).

Notably, hypoxia and inflammation are concomitant events in various pathological immune environments, including chronic inflammation and ischemic tissue (230). Just as hypoxia induces inflammation, inflamed lesions often become severely hypoxic. Under these pathological conditions, tissue hypoxia leads to the activation of the hypoxia-inducible factor (HIF) pathway, which is the master regulator of the adaptive response to hypoxia and directly activates the expression of hundreds of target genes to maintain cellular oxygen homeostasis (231). HIF controls iron regulatory proteins (such as TfR, DMT1, and FPN), suggesting a link between hypoxia and ferroptosis. In diabetes, retina (232), kidney (233), islets (234), fat (235), skin, and wound (236) tissues are all in a state of hypoxia, suggesting that hypoxia plays a central role in the occurrence and development of diabetes and its complications. Hypoxia and inappropriate responses to hypoxia caused by dysregulated HIF-1 signaling are important pathogenic factors in diabetes. In previous studies, in chronic hypoxia-induced diabetes models caused by renal ischemia, HIF-1α and HO-1 levels were found to be increased in the kidney, accompanied by oxidative stress and lipid peroxidation (237). However, these symptoms can be ameliorated by administration of the ferroptosis inhibitor ferrostatin-1. These results suggest that ferroptosis may influence the development of DN through the HIF-1α/HO-1 pathway. Therefore, based on the interrelationship among ferroptosis, inflammation, and hypoxia, regulating HIF pathway activation caused by tissue hypoxia, thereby alleviating the occurrence and development of inflammation and ferroptosis, may be a promising therapeutic mechanism to address diabetes and its complications.

## 5 Summary and prospects

Here, we reviewed the related mechanisms of ferroptosis and potential relationship between ferroptosis and inflammation-related pathways. We subsequently discussed and summarized the relationships among DN, inflammation, and ferroptosis. There is clear evidence that ferroptosis impacts the development of DN; thus, targeted modulation of the ferroptosis process is a potential new therapeutic approach for DN. However, most experiments have aimed to induce or inhibit ferroptosis in tissue cells. Few studies have been conducted on the relationship between inflammation-related pathways and ferroptosis to address disease. Therefore, this review is expected to inform future research.

Iron is an important trace element in the human body, and because iron exists in the body in many forms, its physiological functions are extensive, including oxygen transport, enzyme activity, and energy metabolism. Iron is closely related to energy metabolism; thus, it is abundant in the heart, liver, kidney, and other organs with high physiological activity and extensive biochemical functions (238, 239). Iron balance in the body is maintained through unique systemic and cellular regulation processes. As an important organ in the human body, the kidney plays a key role in maintaining iron balance. Relevant transporters and regulatory pathways involved in iron handling in each kidney cell have been identified (240–246). The IL-6/JAK/STAT3 signaling pathway plays a crucial role in regulating hepcidin expression through the inflammatory response (157). Hepcidin, synthesized and secreted by hepatocytes, acts as the central regulator of iron distribution in different organs. It inhibits intracellular iron export by degrading iron transporter proteins (50). Therefore, inflammation plays a crucial role in maintaining iron balance throughout the body, and its signaling pathways may help regulate iron levels in various organs. Furthermore, there could be a connection between iron metabolism, inflammation, ferroptosis, and kidney disease. Further investigation is needed to confirm these hypotheses and explore this potential association.

In addition to regulating ferroptosis by activating inflammation-related signaling pathways, some inflammatory cells are associated with the occurrence and development of ferroptosis in DN. For example, as phagocytic immune cells, macrophages exist widely in the human body (247). Differentially polarized macrophages play key roles in maintaining homeostasis by mediating inflammation and regulating iron, lipid, and amino acid metabolism through unique functions, such as phagocytosis and exocytosis, cytokine secretion, and ROS production. Several recent studies have implicated macrophages in the pathogenesis of DN. In studies in humans and animal models, macrophages have been found to accumulate in the diabetic kidney, and this accumulation is associated with renal injury in diabetes (248). For example, in diabetic patients, ROS produced in macrophages can induce macrophage polarization to the M1 phenotype, while advanced glycation end products (AGEs) produced by some nonenzymatic glycation reactions can induce ROS production in macrophages and promote polarization to M1 through the RAGE/ROS/TLR1/STAT4 pathway. This mechanism increases the release of a series of inflammatory factors, aggravates inflammation, and provides an opportunity and environment for ferroptosis (249). Furthermore, Liu et al. identified ferroptosis-related genes and pathways in DN through bioinformatics analyses (250). The CIBERSORT algorithm was used explore the relationship between ferroptosis and immune infiltration in DN by evaluating the immune cell infiltration of 22 immune cell gene sets in large sample. The samples were divided into high- and low-risk groups according to the median risk score. In the high-risk group, there was a significant increase in macrophage M2 expression, while neutrophil and mast cell activation showed a significant decrease. Ferroptosis may influence the immune infiltration of M2 macrophages, neutrophils, and activated mast cells in DN (250). In their study on the potential mechanism of ferroptosis-related genes in DN and their relationship with immune inflammatory responses, Ma et al. confirmed that PRDX6 and RGS4, two ferroptosis-related genes, were closely associated with ferroptosis-mediated immune infiltration in DN patients, particularly RGS4 (251). Their findings also suggest that imbalances between driver, suppressor, and marker genes of ferroptosis may be linked to multiple pathogenic factors in kidney disease, primarily involving immunity and inflammation. This finding is crucial for identifying multiple ferroptosis targets for potential interventions (251). Targeting ferroptosis-related immune cell responses, particularly macrophages, may enhance immunotherapy efficacy in DN patients. Additionally, inhibiting macrophage activation/transformation and interactions could be a promising approach to attenuate DN (252, 253). Therefore, given the important role of macrophages in DN and the relationship between macrophages and ferroptosis, exploring the relationships among the three could in further research could be beneficial and help identify new treatments for DN.

An increasing number of studies have shown that inflammation and ferroptosis are closely related to the occurrence and development of DN. However, most studies have mainly focused on ferroptosis in DN and the activation of related inflammatory factors. The specific mechanisms linking ferroptosis and inflammation in DN remain insufficiently understood, and many problems have not been solved. For example, although ferroptosis-related markers and the activation of inflammatory factors can be detected *in vivo* and *in vitro*, confirming the occurrence of ferroptosis and inflammation, diabetic kidney pathology results from interactions among multiple factors. What are the specific factors leading ferroptosis and inflammation in the diabetic kidney, and what are the specific mechanisms of inflammation and ferroptosis in DN? Whether there are specific related pathways and how they interact to drive the development of DN remains unknown. In addition, due to the different functions and distributions of each cell type in the kidney, the specific effects of iron accumulation and other ferroptosis phenomena in the kidney and the potential relationships among these factors and inflammation remain to be explored. Studies on the effect of ferroptosis on DN have revealed some potential compounds and targets, and some drugs have shown good anti-inflammatory and anti-ferroptosis effects in animal models; however, their effectiveness in patients with DN has not been determined. Therefore, in-depth study of the mechanisms associating inflammation, ferroptosis, and DN and the elucidation of their related signaling pathways may provide new molecular targets for the treatment of DN.

## Author contributions

JLL: Writing – original draft. LL: Conceptualization, Writing – original draft. ZZ: Writing – review & editing. PC: Writing – review & editing. HS: Writing – review & editing. CY: Writing – review & editing. YC: Writing – review & editing. JTL: Writing – review & editing.
